# Integrated assessment of heavy metal dynamics, ecological risk, and plant-mediated phytostabilization in mining-affected soils

**DOI:** 10.3389/fpls.2026.1894392

**Published:** 2026-07-09

**Authors:** Dias Daurov, Kabyl Zhambakin, Ainash Daurova, Zagipa Sapakhova, Iskander Isgandarov, Zhanar Abilda, Zhanibek Sunatullayev, Igor Klein, Assem Vyrakhmanova, Raushan Ramazanova, Aidar Sumbembayev, Malika Shamekova

**Affiliations:** 1 Laboratory of Breeding And Biotechnology, Institute of Plant Biology and Biotechnology, Almaty, Kazakhstan; 2 Tanir Research Laboratory, Almaty, Kazakhstan; 3 Faculty of Agrobiology, Kazakh National Agrarian Research University, Almaty, Kazakhstan; 4 German Remote Sensing Data Center (DFD), German Aerospace Center (DLR), Wessling, Germany; 5 U.U. Uspanov Kazakh Research Institute of Soil Science and Agrochemistry, Almaty, Kazakhstan; 6 Altai Botanical Garden, Ridder, Kazakhstan

**Keywords:** Belousovka mining area, East Kazakhstan Region, ecological risk, heavy metals, physicochemical properties, pollution indices, soil contamination

## Abstract

**Introduction:**

Mining-affected soils are complex geochemical systems characterized by long-term heavy metal accumulation, ecological degradation, and altered vegetation structure. Understanding the interactions among soil properties, contaminant mobility, ecological risk, and plant responses is essential for evaluating remediation potential in post-mining ecosystems. This study provides the first integrated assessment of soil properties, ecological risk, vegetation structure, landscape heterogeneity, and plant-mediated phytostabilization in the Belousovka mining area.

**Methods:**

Soil physicochemical properties, granulometric composition, heavy metal concentrations, pollution indices, multivariate statistics, spatial analysis, and plant accumulation characteristics were investigated at 17 representative sampling sites, including two reference locations. Concentrations of Zn, Cu, Cd, and Pb were determined in soils and in root and shoot tissues of *Helianthus annuus* L. and *Triticum aestivum* L., two dominant agricultural crops representative of local agroecosystems, using 12 plant samples collected from two representative contaminated sites selected for detailed plant accumulation assessment. Pollution Load Index (PLI), Potential Ecological Risk Index (RI), bioconcentration factor (BCF), and translocation factor (TF) were calculated.

**Results:**

Zn and Cu were the dominant contaminants (1641 and 205 mg·kg^−^¹, respectively), whereas Cd represented the principal ecological risk factor. Severe localized contamination was observed (PLI = 74.67; RI = 3992.46). PERMANOVA confirmed significant differences among ecological risk groups (R² = 0.72, p = 0.001). Heavy metal distribution was strongly influenced by soil texture, pH, carbonate content, and terrain-related redistribution processes. Vegetation surveys revealed dominance of ruderal, perennial, and phytostabilizing species in industrial areas, indicating ongoing spontaneous succession. *Helianthus annuus* L. exhibited substantially greater biomass and metal accumulation capacity than *Triticum aestivum* L., particularly for Zn, Cu, and Cd. Root tissues consistently accumulated higher metal concentrations than shoots, with TF values predominantly below 1.

**Discussion:**

Ecological risk was controlled by technogenic contamination and soil properties, whereas predominant root sequestration indicated phytostabilization as the principal adaptive response under contaminated conditions.

## 1 Introduction

Mining and metallurgical activities are widely recognized as major sources of long-term environmental contamination due to the large-scale release and redistribution of potentially toxic elements within terrestrial ecosystems (Mourinha et al., 2022). Among the various pollutants associated with technogenic landscapes, heavy metals (HMs) are of particular concern because of their persistence, non-biodegradable nature, capacity for bioaccumulation, and potential ecological and human health impacts (Briffa et al., 2020; Das et al., 2023; Daurov et al., 2023). Soil systems located near active or abandoned mining areas frequently act as long-term geochemical sinks for metal-bearing particles derived from ore extraction, tailing weathering, smelting emissions, and contaminated runoff. As a result, mining-affected soils commonly exhibit substantial alterations in physicochemical properties, nutrient balance, and biological functioning (Toishimanov et al., 2023).

The environmental behavior of heavy metals in soils is controlled not only by the intensity of anthropogenic inputs but also by the physicochemical characteristics of the soil matrix itself. Parameters such as pH, carbonate content, particle-size distribution, organic matter, and exchangeable cation composition strongly influence metal mobility, adsorption, precipitation, and ecological availability (Daurov et al., 2026; Smičiklas et al., 2015). Fine-textured soils enriched in clay and silt fractions generally demonstrate greater sorption capacity due to their higher specific surface area and abundance of reactive mineral phases, whereas coarse-textured substrates often promote enhanced migration and redistribution of contaminants (Button et al., 2022). Similarly, carbonate-rich soils may reduce the mobility of certain trace metals through precipitation and buffering processes, while acidic environments typically enhance metal solubility and bioavailability (Khan et al., 2025). Therefore, understanding the interactions between soil properties and heavy metal accumulation is essential for accurate ecological risk assessment in mining-affected environments (Li et al., 2025).

Eastern Kazakhstan is one of the largest mining and metallurgical regions of Central Asia and contains numerous polymetallic deposits associated with Zinc (Zn), Lead (Pb), Copper (Cu), Cadmium (Cd), and other sulfide-bearing ores. Intensive industrial development during the Soviet and post-Soviet periods resulted in extensive environmental transformation across many mining districts of the East Kazakhstan Region (EKR). Long-term ore extraction, processing, and tailing storage contributed to the accumulation of heavy metals in soils, sediments, surface waters, and vegetation. In addition to geochemical contamination, long-term mining activity may substantially alter ecosystem structure, vegetation cover, and habitat stability within post-mining landscapes. Changes in land use and land cover (LULC), combined with technogenic soil transformation, can affect biodiversity conservation, vegetation succession, nutrient cycling, and ecosystem resilience. Mining-related disturbance frequently leads to fragmentation of natural habitats, reduction of vegetation density, and redistribution of fine contaminated sediments, thereby influencing both terrestrial and riparian ecological communities. Several industrial centers of the region, including Ridder, Ust-Kamenogorsk, Zyryanovsk, and Belousovka, are considered environmentally vulnerable territories because of prolonged technogenic pressure and insufficient remediation of historical mining infrastructure (Dyussembayeva et al., 2025).

The Belousovka mining area, located within the Altai metallogenic province of East Kazakhstan, represents one of the historically important polymetallic mining territories of the region. The area is characterized by complex geological structure and widespread occurrence of sulfide mineralization dominated by sphalerite, chalcopyrite, galena, and associated ore minerals. Mining activity in Belousovka has been conducted for decades and included underground ore extraction, beneficiation processes, and disposal of mining wastes. As in many abandoned or partially inactive mining regions, long-term weathering of ore-bearing material and tailings potentially contributes to continuous release of Zn-, Cu-, Cd-, and Pb-bearing particles into surrounding ecosystems (Wang et al., 2019). However, despite the recognized industrial significance of Belousovka, comprehensive environmental investigations of soil geochemistry and ecological risk remain comparatively limited relative to other mining districts of East Kazakhstan. Recently, increasing attention has been devoted not only to contamination intensity but also to the ecological functioning of post-mining ecosystems and adaptive responses of vegetation under heavy metal stress. Plant species growing in contaminated soils may substantially differ in biomass production, metal accumulation, and translocation behavior depending on their physiological tolerance and root sequestration mechanisms. In particular, *Helianthus annuus* L. (*H. annuus*) has frequently been identified as a promising phytostabilization species because of its high biomass productivity and strong capacity for root accumulation of Zn, Cu, and Cd, whereas *Triticum aestivum* L. (*T. aestivum)* generally demonstrates lower accumulation potential under contaminated conditions (Khalid et al., 2018; Paulo et al., 2023). The investigation of bioconcentration factor (BCF) and translocation factor (TF) values therefore provides important insight into contaminant mobility within plant tissues and ecological stabilization processes in technogenically transformed landscapes.

Despite increasing attention to environmental contamination in mining regions, many studies remain focused primarily on total metal concentrations, whereas considerably less attention has been devoted to the combined influence of soil physicochemical properties and granulometric composition on contaminant distribution and ecological risk (Kolawole et al., 2018; Barakat et al., 2021). In addition to soil contamination, mining activity substantially alters vegetation structure, biodiversity, and ecological succession processes within disturbed landscapes. Spontaneous establishment of ruderal, perennial, and metal-tolerant plant species may contribute to natural phytostabilization and ecological recovery of contaminated substrates. In particular, perennial grasses, legumes, and pioneer woody species are considered important components of vegetation-mediated stabilization in post-mining ecosystems. However, the mobility and ecological significance of heavy metals are strongly dependent on soil texture, carbonate buffering, organic matter accumulation, and exchangeable cation composition. For example, fine-textured soils may enhance sorption and immobilization of metals due to their high surface area, whereas sandy substrates can facilitate contaminant migration and redistribution. Similarly, carbonate-rich soils may partially reduce metal mobility through precipitation and adsorption processes. Therefore, integrated investigations combining geochemical, physicochemical, and ecological approaches are essential for understanding contaminant behavior in post-mining environments (Azizi et al., 2025).

In recent years, pollution assessment indices such as the contamination factor (Cf), geoaccumulation index (Igeo), Pollution Load Index (PLI), and Potential Ecological Risk Index (RI) have become widely applied tools for evaluating anthropogenic enrichment and ecological hazard in contaminated soils (Altin et al., 2026, da Silva Junior et al., 2022). These indices provide complementary information by distinguishing cumulative contamination intensity from toxicological risk associated with individual metals. Multivariate statistical approaches, including redundancy analysis (RDA), non-metric multidimensional scaling (NMDS), and hierarchical clustering, additionally allow identification of relationships between environmental variables, contamination gradients, and ecological risk structure (Muniz and Oliveira-Filho, 2023).

Despite the growing number of studies addressing heavy metal contamination in mining environments, significant knowledge gaps remain regarding the integrated influence of soil physicochemical properties, granulometric composition, vegetation structure, and landscape heterogeneity on contaminant distribution and ecological risk. Most previous investigations have primarily focused on contamination levels and pollution indices, whereas considerably less attention has been devoted to the interactions among soil characteristics, spontaneous vegetation succession, and plant-mediated stabilization processes in mining-affected ecosystems. Furthermore, comprehensive studies combining geochemical assessment, ecological risk evaluation, biodiversity analysis, terrain characteristics, and phytostabilization potential within a unified analytical framework remain comparatively limited, particularly in Central Asia. This study represents the first integrated assessment of the Belousovka mining area that simultaneously evaluates soil contamination, ecological risk, vegetation structure, land cover heterogeneity, and heavy metal accumulation in dominant agricultural crops. Such an approach provides a broader understanding of ecosystem functioning, contaminant dynamics, and natural recovery processes in post-mining landscapes. We hypothesized that soil physicochemical properties, particularly texture, pH, carbonate content, and exchangeable cation composition, represent the principal factors controlling heavy metal distribution, ecological risk patterns, and plant accumulation behavior within the Belousovka mining landscape. Furthermore, we hypothesized that spontaneous vegetation communities and dominant agricultural crops would exhibit adaptive responses to contamination through metal immobilization and phytostabilization mechanisms.

Therefore, the present study aimed to provide a comprehensive assessment of soil physicochemical properties, granulometric composition, heavy metal contamination, ecological risk, and plant accumulation characteristics in the Belousovka mining area of Eastern Kazakhstan. Particular attention was devoted to evaluating the relationships between soil properties, terrain heterogeneity, heavy metal distribution, and plant accumulation behavior of *Helianthus annuus* L. and *Triticum aestivum* L. using integrated geochemical, ecological, and multivariate statistical approaches. The study contributes to understanding contaminant dynamics, phytostabilization potential, and ecological functioning within technogenically transformed post-mining ecosystems.

## 2 Materials and methods

### 2.1 Study area

**Figure 1:**
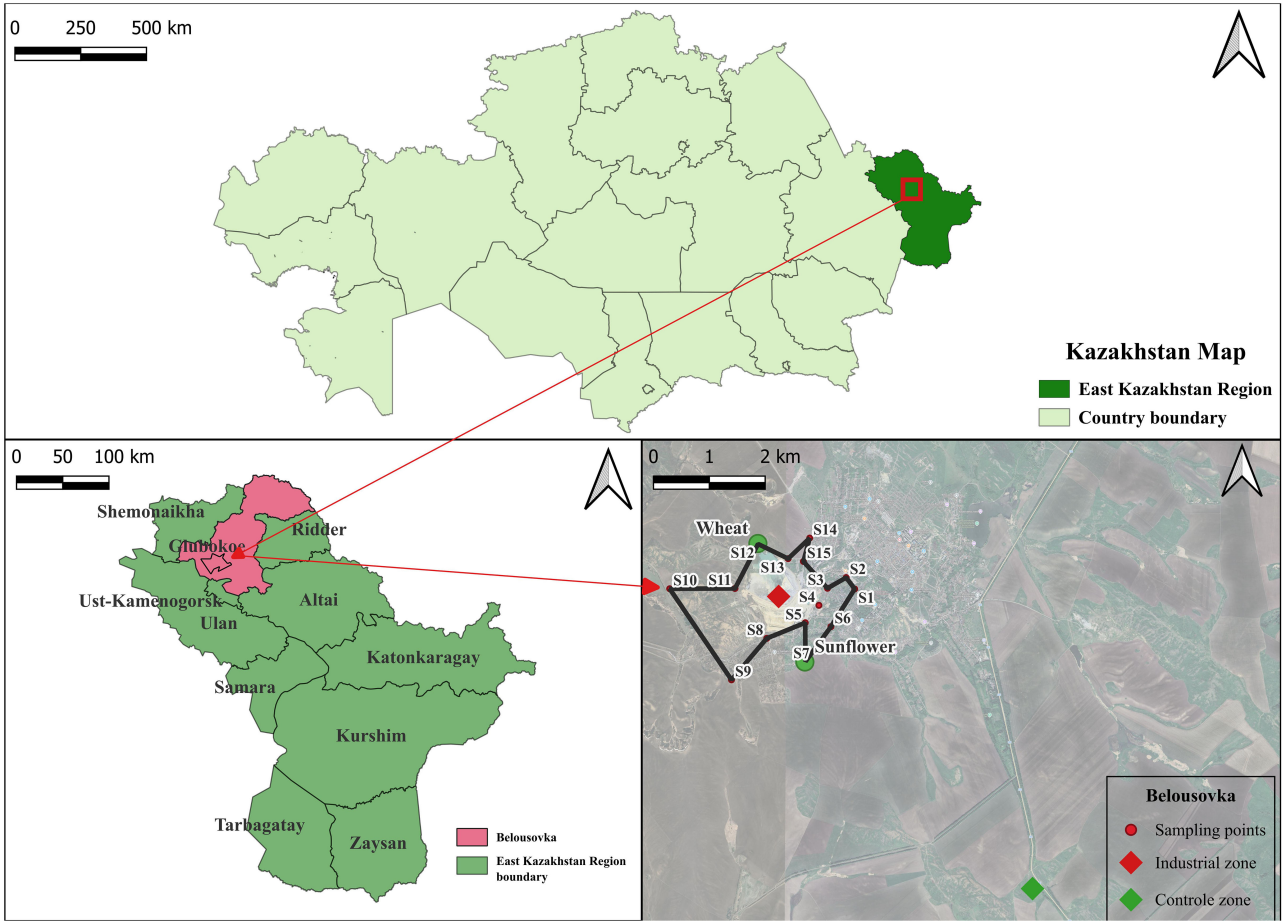
Distribution of the sample locations in the study area.

The study was conducted in the Belousovka mining area located in the East Kazakhstan Region of the Republic of Kazakhstan (**Figure 1**). Belousovka (50°07′44′′ N, 82°30′39′′ E) is an urban-type settlement situated in the Glubokovsky District, approximately 14 km east of Glubokoe and 11 km north of Ust-Kamenogorsk. The settlement is located within one of the historically important polymetallic mining regions of Kazakhstan and has long been associated with intensive mining and ore-processing activities related primarily to the extraction of zinc, cadmium, copper, lead, and other non-ferrous metals (**Supplementary Figure 1**).

The study area is influenced by former and active mining infrastructure, ore-processing facilities, tailing storage sites, and waste disposal zones, which represent the principal anthropogenic sources of potentially toxic elements entering the surrounding environment. Belousovka is situated within the mountainous and foothill landscapes of the Altai geological province, where heterogeneous topography affects the atmospheric transport, redistribution, and deposition of contaminated particles.

**Figure 2:**
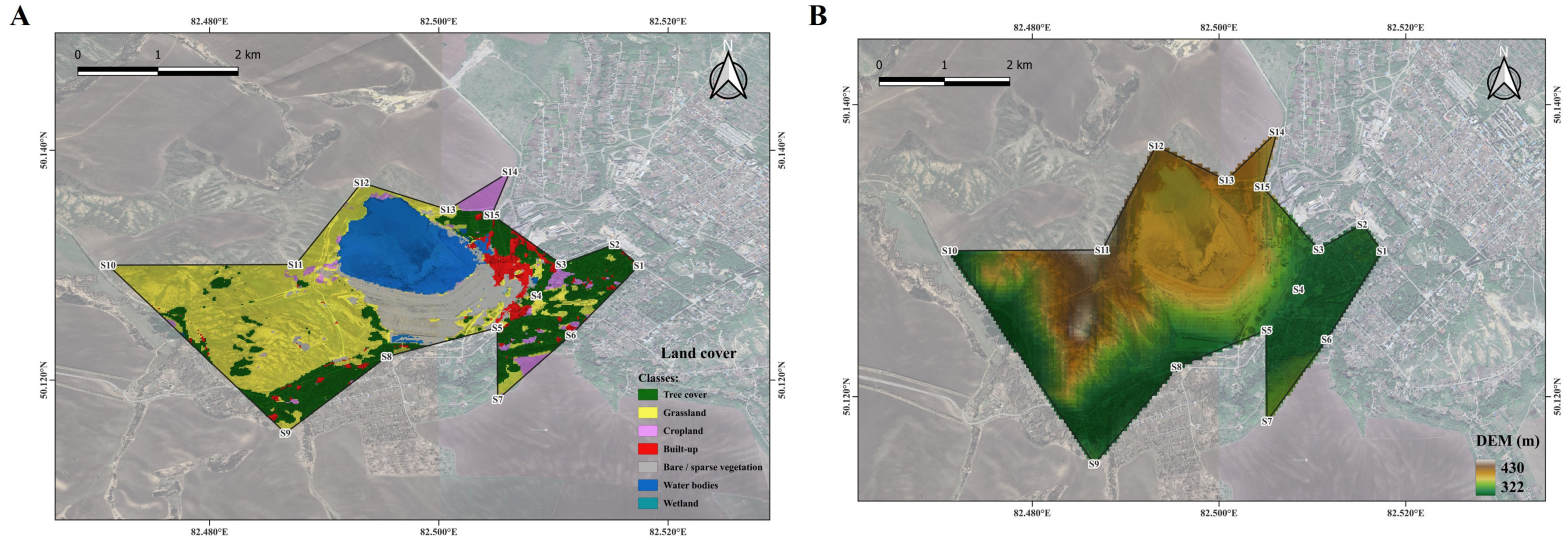
Environmental characteristics of the Belousovka mining area: **(A)** land use/land cover (LULC) classification and **(B)** digital elevation model (DEM).

The climate of the region is sharply continental, characterized by cold winters, moderately warm summers, and pronounced seasonal temperature fluctuations. Average annual precipitation ranges from 600 to 700 mm, associated with active geochemical migration processes, including leaching, surface runoff, and secondary redistribution of contaminants. The study area is characterized by foothill landscapes with fragmented terrain and mixed geomorphological conditions that may influence surface runoff and spatial redistribution processes. Land use/land cover analysis derived from the European Space Agency (ESA) WorldCover 2021 dataset indicated the predominance of urbanized and agricultural landscapes surrounding the mining-affected zone (**Figure 2A**). The Belousovka mining district is additionally associated with carbonate-containing parent materials and polymetallic ore mineralization, which influence soil buffering capacity, contaminant retention, and heavy metal mobility. Variable soil texture may further regulate heavy metal sorption and ecological behavior within mining-affected landscapes. The LULC analysis additionally revealed a heterogeneous ecological mosaic dominated by grasslands, tree-covered areas, agricultural lands, wetlands, and technogenically disturbed surfaces surrounding the former mining infrastructure. Semi-natural vegetation patches may partially stabilize contaminated soils through reduction of surface runoff, enhancement of soil aggregation, and limitation of aeolian transport of fine particles. In contrast, bare and sparsely vegetated areas located near industrial zones potentially represent ecologically vulnerable hotspots characterized by reduced biological productivity and increased susceptibility to contaminant migration. The coexistence of natural and anthropogenically transformed landscapes indicates substantial spatial heterogeneity in habitat conditions and ecological functioning within the Belousovka mining district.

Topographic analysis based on the Copernicus digital elevation model (DEM) GLO-30 dataset demonstrated moderate elevation variability and heterogeneous relief conditions within the investigated territory (**Figure 2B**). The observed topographic heterogeneity may substantially influence hydrological redistribution, erosion intensity, sediment transport, and localized accumulation of contaminated particles within the mining landscape. Lower geomorphological positions may function as depositional zones for fine technogenic sediments, whereas elevated and exposed areas are more susceptible to erosion and secondary contaminant redistribution. Such terrain-controlled processes are particularly important in post-mining ecosystems because they affect both contaminant mobility and vegetation recovery dynamics.

### 2.2 Soil sampling and physicochemical analysis

Soil sampling was carried out using the envelope (diagonal composite) method, in which five subsamples were collected diagonally across each sampling plot and combined into a composite sample. A total of 17 sampling sites were investigated across the Belousovka mining area. The sampling sites were selected using a targeted environmental gradient approach to represent different levels of mining influence and environmental heterogeneity within the study area. Site selection considered proximity to historical mining and ore-processing facilities, land cover characteristics, terrain variability, and the spatial distribution of potential contamination sources identified during preliminary field reconnaissance and GIS-based analysis. The selected sites encompassed highly disturbed industrial zones, intermediate transition areas, and relatively less impacted locations, thereby allowing assessment of contamination gradients and associated ecological responses across the mining landscape. Although no pristine reference sites were available within the mining district, sites S16 and S17, located at greater distances from historical mining and ore-processing facilities and characterized by the lowest pollution index values, were considered relatively low-impact reference locations for comparative ecological assessment. Soil samples were collected from a depth of 20–50 cm in order to evaluate the long-term vertical migration and redistribution of heavy metals within subsurface soil horizons and to minimize the influence of recent surface deposition processes. This depth interval was selected because it represents a relatively stable soil layer that better reflects historical contamination associated with prolonged mining activity and provides insight into contaminant retention, mobility, and ecological risk within the root-influenced zone. The geographic coordinates of the sampling sites were recorded using a global positioning system (GPS) device. Mesotopography, functional zone, and soil classification according to the World Reference Base for Soil Resources (WRB) system were determined based on field observations, geomorphological characteristics, and physicochemical soil properties. Detailed environmental characteristics of the sampling sites are presented in **Supplementary Table 1**. The mass of each individual subsample did not exceed 200 g, while the total mass of each composite sample was at least 1 kg, ensuring its representativeness for subsequent laboratory analyses. Following transportation to the laboratory, soil samples were air-dried under ambient conditions, manually cleaned from visible plant residues and coarse debris, and subsequently homogenized. The dried material was sieved through a 2 mm nylon mesh to obtain the fine-earth fraction used for physicochemical and geochemical analyses.

Physicochemical properties of soil samples were determined using standard analytical procedures widely applied in environmental soil science and geochemical investigations. Prior to analysis, soil samples were air-dried, homogenized, and sieved through a 2 mm mesh. Soil pH was measured potentiometrically using a laboratory pH meter (SevenCompact S220, Mettler Toledo, Greifensee, Switzerland) in a 1:2.5 soil-to-1 M KCl suspension. Total organic carbon (TOC) content was determined by the dichromate oxidation method followed by titrimetric quantification (Walkley and Black, 1934). The analysis was based on oxidation of organic carbon using potassium dichromate (K_2_Cr_2_O_7_) in concentrated sulfuric acid (H_2_SO_4_), with the excess dichromate determined by titration with ferrous sulfate solution using standard laboratory titration equipment (automatic burette system, Brand GmbH,Wertheim, Germany) (Arinushkina, 1992). Total nitrogen (N) was measured using the Kjeldahl digestion method (Kjeldahl, 1883). Available phosphorus was determined with a spectrophotometer by extracting 0.5 M NaHCO_3_ (pH 8.5) (Olsen et al., 1954). Exchangeable potassium (K), calcium (Ca), and magnesium (Mg) were extracted with ammonium acetate (AAc) (1 M, pH 7.00). Soil texture was determined using the Kachinsky pipette sedimentation method, a classical particle-size analysis technique based on the sedimentation principle according to Stokes’ law, allowing the quantification of sand, silt, and clay fractions (Kachinskiy, 1965).

A quantity of 2 g of dried soil sample was transferred into a 100 mL Erlenmeyer flask and concentrated nitric acid (65%, analytical grade) was added. Heavy metals were extracted using a nitric acid extraction procedure widely employed in environmental geochemical and soil contamination studies (Willett and Zarcinas, 1986). Then, the sample was boiled in a water bath at 80 °C for 3 h. Next, it was cooled at room temperature, 25 mL water was added, and the resulting extract was filtered on filter paper into a 25 mL volumetric flask. These clear filtrate solutions were analysed using an atomic absorption spectrometer. Concentrations of Pb, Zn, Cd, and Cu were determined by flame atomic absorption spectrometry (AAS). HM concentrations in polluted soils exceeding the maximum permissible concentration (MPC) were determined according to the regulations. MPC values (mg·kg^–1^) for HMs were 32.0 for Pb, 23.0 for Zn, 2.0 for Cd and 3.0 for Cu. The applied MPC thresholds correspond to environmental regulatory standards commonly used for assessing heavy metal contamination in soils of Kazakhstan and neighboring post-Soviet mining regions (Federal Center for Hygiene and Epidemiology of Rospotrebnadzor, 2006).

### 2.3 Plant sampling and heavy metal determination

Plant sampling was conducted simultaneously with soil sampling within the Belousovka mining area in order to evaluate heavy metal accumulation, translocation, and vegetation adaptation under mining-related contamination conditions. Two dominant cultivated plant species widely distributed within the investigated territory, *Helianthus annuus* L. and *Triticum aestivum* L., were selected as model species for ecological assessment because of their contrasting physiological characteristics and potential differences in heavy metal uptake behavior. To additionally assess the natural biodiversity of the study area, geobotanical surveys were conducted at sites located near industrial facilities as well as at control locations situated at a distance from major sources of industrial impact. Vegetation sampling was performed during the peak vegetation period (June–July) using standard geobotanical survey approaches widely applied in ecological studies of mining-affected landscapes. Representative 1 × 1 m plots were established at each sampling site to characterize vascular plant diversity, vegetation structure, and ecological differentiation between technogenically disturbed and relatively undisturbed habitats. One 1 × 1 m quadrat was established at each sampling site, resulting in a total surveyed area of 17 m² across the study area. Plant species were additionally classified according to their life forms (annuals, perennial herbs, shrubs, and woody pioneer species). The Importance Value Index (IVI) was calculated as the sum of relative abundance, relative frequency, and relative coverage for each species. Projective cover (%) was estimated proportionally from the Importance Value (IV) index and expressed as the relative percentage contribution of each species within the plant community.

Representative mature plants of *H. annuus* and *T. aestivum* were collected from sampling sites characterized by varying contamination intensity. At each site, three biological replicates were collected for each species. Whole plants were carefully excavated together with the root system in order to minimize mechanical damage and loss of fine roots. The collected material was separated into aboveground (shoot) and belowground (root) fractions immediately after sampling.

Plant roots were thoroughly washed with tap water followed by rinsing with deionized water to remove adhered soil particles and surface contaminants. Shoot samples were also rinsed with deionized water to eliminate atmospheric dust deposition. All plant materials were subsequently dried at 65–70 °C to constant weight under laboratory conditions. Dry biomass was determined gravimetrically and expressed as g·plant^−^¹. The dried samples were homogenized using a laboratory grinder and stored in clean polyethylene containers prior to chemical analysis.

For heavy metal determination, dried plant material was subjected to acid digestion using concentrated nitric acid (HNO_3_) and hydrogen peroxide (H_2_O_2_) according to standard procedures commonly applied in environmental and agricultural studies. After digestion, concentrations of Zn, Cu, Cd, and Pb in root and shoot tissues were determined using atomic absorption spectrometry (AAS). Heavy metal concentrations were expressed on a dry weight (DW) basis as mg·kg^−^¹ DW. Quality assurance and quality control (QA/QC) procedures included analysis of reagent blanks, replicate measurements, calibration verification, and recovery testing using certified reference standard solutions. Calibration curves demonstrated excellent linearity (R² = 0.9995–0.9997). Method accuracy was evaluated using certified reference solutions, yielding recoveries ranging from 99.1% to 100.3%, while analytical precision was confirmed by low standard deviations obtained from replicate analyses. Limits of detection (LOD) and limits of quantification (LOQ) ranged from 0.04 to 0.08 mg·L^−^¹ and from 0.12 to 0.24 mg·L^−^¹, respectively. Detailed calibration parameters, recovery data, LOD, and LOQ values are provided in **Supplementary Tables 2** and **3**.

### 2.3.1 Bioconcentration and translocation factors

To evaluate heavy metal accumulation and translocation characteristics, the bioconcentration factor (BCF) and translocation factor (TF) were calculated according to the following equations:


$$BCF=\frac{C_{plant}}{C_{soil}}$$

$$TF=\frac{C_{shoot}}{C_{root}}$$

where *C_plant_* represents heavy metal concentration in plant tissues (root or shoot), *C_soil_* is the corresponding heavy metal concentration in soil, *C_shoot_* is the concentration in aboveground tissues, and *C_root_* is the concentration in roots.

BCF values were used to evaluate the capacity of plants to accumulate metals from contaminated soils, whereas TF values characterized the efficiency of metal translocation from roots to aerial tissues. Values of TF< 1 were interpreted as predominant root sequestration and limited upward translocation, indicating phytostabilization potential under contaminated environmental conditions.

### 2.4 Atomic absorption spectrometry

Calibration curves were constructed for lead (Pb), zinc (Zn), cadmium (Cd), and copper (Cu) using certified atomic absorption stock solutions (1.0 g/L; Agilent Technologies, Santa Clara, CA, USA), prepared by successive dilution. Working calibration standards in the concentration range of 1–10 mg·L^–1^ were obtained using ultrapure water produced by a Milli-Q purification system (Merck Millipore, Darmstadt, Germany). All reagents were of analytical grade, including nitric acid (65%) and hydrogen peroxide (30%).

The analytical procedure was validated using reference standard solutions, with acceptable results required to fall within ± 1% of the certified values. Method validation included the evaluation of linearity and calibration range. Linearity was assessed by analyzing reference standard solutions in five independent replicates for each element. The limits of detection (LOD) and limits of quantification (LOQ) for Pb, Zn, Cd, and Cu were determined based on measurements at concentrations of 1, 2, 5, and 10 mg·L^–1^.

The concentrations of Pb, Zn, Cd, and Cu were determined using a flame atomic absorption spectrometer (Agilent 240FS, Agilent Technologies, Santa Clara, CA, USA) equipped with single-element hollow cathode lamps and an air-acetylene flame. The analytical wavelengths were 283.3 nm for Pb, 232.0 nm for Zn, 228.8 nm for Cd and 324.8 nm for Cu. The gas flow rate was maintained at 50 dm^3^/h, with an aspiration rate of 5 cm^3^/min. Single-element hollow cathode lamps (Agilent Technologies, Santa Clara, CA, USA) specific to each metal were used as radiation sources (**Supplementary Table 2**).

### 2.4.1 Validation of the method

Quantitative determination of heavy metals (HMs) was performed using external calibration, with calibration curves constructed over the concentration range of 1.0–10.0 mg·L^–1^. Quality assurance and quality control (QA/QC) procedures included procedural blanks, duplicate analyses, calibration verification standards, and recovery tests using certified reference standard solutions. Each calibration curve was generated using four concentration levels. The calibration parameters, including correlation coefficients (R^2^), regression equations, linear ranges, limits of detection (LOD), and limits of quantification (LOQ) for each HM, are summarized.

All calibration curves exhibited excellent linearity, with correlation coefficients exceeding 0.9995, indicating a strong analytical response across the tested concentration range. The LOD values ranged from 0.04 to 0.08 mg·L^–1^, while the LOQ values varied between 0.12 and 0.24 mg·L^–1^, demonstrating the high sensitivity and suitability of the method for trace-level determination of heavy metals.

**Supplementary Table 3** presents the mean recovery values and corresponding standard deviations obtained using certified reference standard solutions for each heavy metal. The measured concentrations showed good agreement with the certified values at the 95% confidence level, confirming the accuracy of the analytical method. The mean recovery values ranged from 99.1% to 100.3% across all analysed metals. Analytical precision was further evaluated through duplicate analyses, and relative standard deviation (RSD) values were below 5% for all analysed metals, indicating satisfactory repeatability, precision, and reliability of the analytical method.

### 2.4.2 Sample digestion

Approximately 0.5 g of homogenized sample was placed in a digestion vessel and treated with 5 mL of concentrated nitric acid (65%, analytical grade). The mixture was pre-digested at room temperature for 30 min and subsequently heated on a digestion block at 120 °C until near dryness. After cooling, 2 mL of hydrogen peroxide (30%) was added to complete oxidation of organic matter. The digest was diluted to a final volume of 25 mL with ultrapure water (Milli-Q system, Merck Millipore, Germany), filtered, and analysed by flame atomic absorption spectrometry. The procedure followed a modified nitric acid–hydrogen peroxide digestion protocol commonly applied for environmental soil analysis.

### 2.5 Pollution assessment and ecological risk evaluation

To comprehensively evaluate soil contamination and ecological risk associated with heavy metal accumulation, several widely used geochemical and ecological indices were calculated.

The contamination factor (CF) was used to evaluate the degree of contamination for individual metals and was calculated as:


$$CF=\frac{C_{n}}{B_{n}}$$

where *C_n_* is the measured concentration of the metal in soil and *B_n_* represents its geochemical background concentration.

The geoaccumulation index (Igeo) was calculated according to the equation proposed by Müller (Müller, 1979):


$$Igeo=log_{2\left(\frac{C_{n}}{1.5B_{n}}\right)}$$

where the constant factor 1.5 compensates for natural lithogenic variability and minor background fluctuations (**Supplementary Table 4**).

The ecological risk factor (Er) was determined as:


$$Er=T_{r}\times CF$$

where *T_r_* denotes the toxic-response coefficient specific to each heavy metal. The toxic-response coefficients used in ecological risk calculations were 30 for Cd, 5 for Cu and Pb, and 1 for Zn according to Hakanson (**Supplementary Table 5**) (Hakanson, 1980).

The integrated pollution load index (PLI) was calculated to evaluate the overall level of contamination across all investigated metals:


$$PLI={\left(CF_{1}\times CF_{2}\times \ldots \times CF_{n}\right)}^{\frac{1}{n}}$$

Potential ecological risk index (RI) values were calculated as the cumulative sum of ecological risk factors:


RI=∑Er

Although the Hakanson method was originally developed for aquatic sediments, the RI and Er indices have been widely applied in studies of contaminated soils, including mining-affected environments, because they integrate contamination intensity and metal-specific toxicological response factors, thereby providing a useful comparative measure of potential ecological risk. Because reliable local geochemical background values are not available for the Belousovka mining district and pristine soils unaffected by historical mining activities are difficult to identify, background concentrations used for calculating the contamination factor (Cf), geoaccumulation index (Igeo), Pollution Load Index (PLI), and Potential Ecological Risk Index (RI) were derived from the least impacted reference sites (S16 and S17). These sites were located outside the principal zone of mining influence and exhibited the lowest heavy metal concentrations and pollution index values within the study area.

### 2.6 Statistical analyses

Statistical analyses and graphical visualizations were performed in the R statistical environment (version 4.6.0), while spatial interpolation and mapping were conducted in QGIS (version 3.40.7). Prior to statistical analyses, quantitative variables were examined for consistency and standardized where appropriate to minimize scale effects among environmental parameters and heavy metal concentrations. Prior to redundancy analysis (RDA), variance inflation factors (VIFs) were calculated to assess multicollinearity among explanatory variables. Variables with VIF values > 10 were considered highly collinear and were excluded from subsequent analyses.

Descriptive statistical analyses were used to characterize the distribution and variability of soil physicochemical properties and heavy metal concentrations. Data distributions and variability patterns were visualized using boxplots and violin plots generated with the *ggplot2* package (version 4.0.3). Soil texture composition was additionally illustrated using ternary diagrams constructed with the *ggtern* package (version 4.0.0).

Relationships among soil physicochemical properties, heavy metals, and pollution indices were evaluated using Spearman rank correlation analysis implemented in the *Hmisc* package (version 5.2–5), with statistical significance accepted at p< 0.05. Correlation matrices and hierarchical clustering heatmaps were generated using Z-score standardized data. Hierarchical clustering was performed based on Euclidean distance matrices and Ward’s linkage method using the *pheatmap* and *stats* packages (version 1.0.13 and 4.6.0).

Multivariate relationships between soil physicochemical properties and heavy metal contamination were investigated using redundancy analysis (RDA) implemented in the *vegan* package (2.7–3). The significance of the RDA model and canonical axes was evaluated using Monte Carlo permutation tests with 999 permutations. Adjusted coefficient of determination (R²) values were additionally calculated to estimate the proportion of variance explained by environmental variables after correction for model complexity.

Non-metric multidimensional scaling (NMDS) based on Bray–Curtis dissimilarity matrices was used to visualize similarities and differences among sampling sites according to heavy metal contamination patterns. The NMDS solution was computed with up to 999 random starts (trymax = 999) to reduce the risk of convergence to local minima and to ensure solution stability. Ordination quality was evaluated using stress values. Differences among ecological risk groups were tested using permutational multivariate analysis of variance (PERMANOVA) with 999 permutations. Homogeneity of multivariate dispersion among groups was evaluated prior to PERMANOVA interpretation to ensure that observed differences reflected variation among groups rather than differences in within-group variability.

Mantel tests were performed to evaluate multivariate associations between soil physicochemical properties, heavy metals, and integrated pollution indices. Significant relationships identified by Mantel analysis were visualized using Mantel correlation networks.

Spatial distribution maps of heavy metals were generated using inverse distance weighting (IDW) interpolation in QGIS. Interpolation performance was evaluated using cross-validation procedures based on prediction errors and root mean square error (RMSE) statistics to assess the reliability of the spatial models. Interpolation was constrained to the polygon representing the study area in order to improve the spatial representation and readability of contamination patterns.

## 3 Results

### 3.1 Soil texture characteristics and physicochemical properties

**Figure 3:**
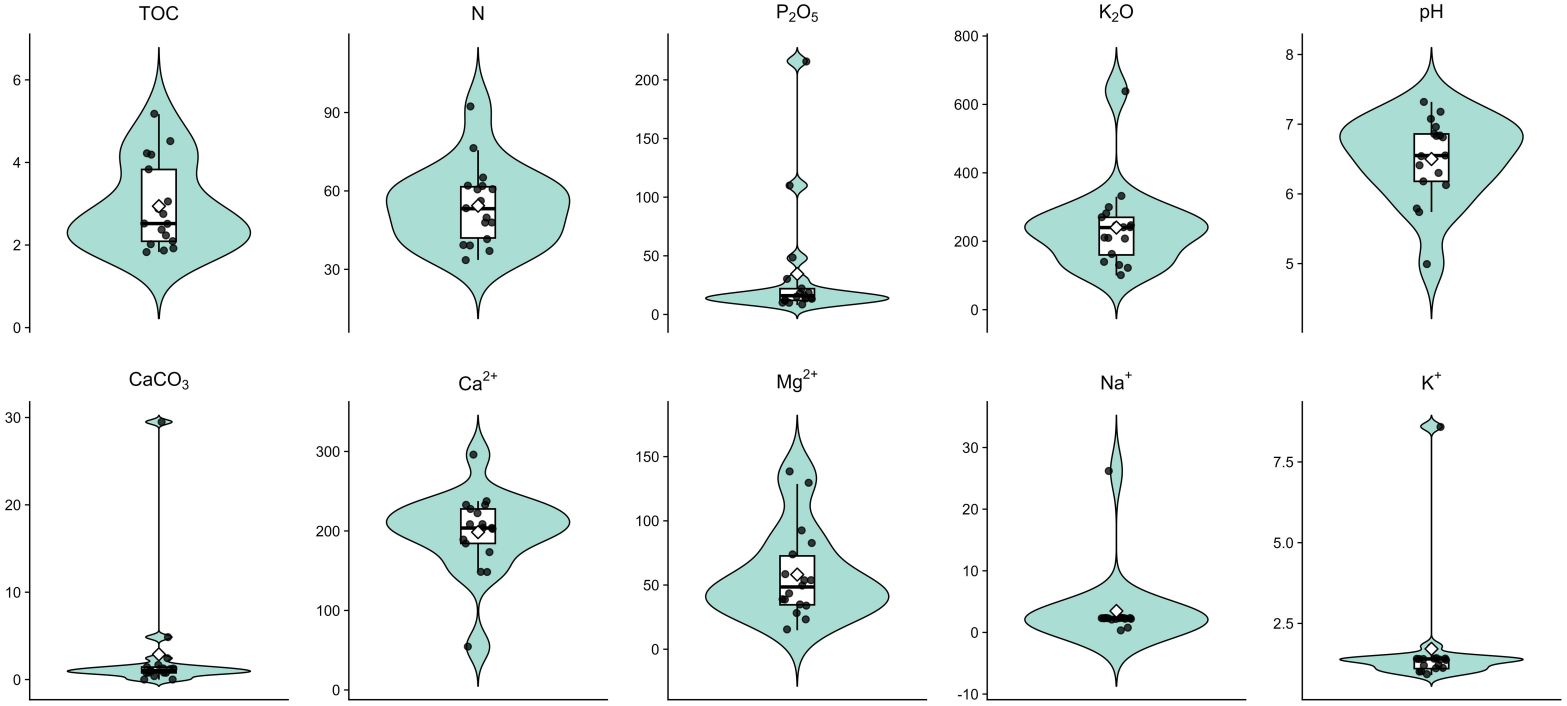
Distribution and variability of soil physicochemical properties in the Belousovka mining area revealed by violin-boxplot analysis.

The investigated soils demonstrated substantial physicochemical heterogeneity associated with lithological variability and long-term mining influence in the Belousovka area (**Figure 3**). Considerable differences were observed in nutrient status, buffering capacity, and exchangeable base composition across the study area (**Supplementary Table 6**).

Total organic carbon (TOC) ranged from 1.83% to 5.17%, while available nitrogen varied between 33.6 and 92.4 mg·kg^−^¹. Both parameters showed relatively moderate variability and similar distribution patterns, indicating close relationships between soil organic matter accumulation and nitrogen retention processes. In contrast, available phosphorus (P_2_O_5_) and exchangeable potassium (K_2_O) exhibited substantially greater heterogeneity, ranging from 8–216 and 100–640 mg·kg^−^¹, respectively. Their strongly skewed distributions indicate localized nutrient enrichment and substantial spatial heterogeneity.

Soil pH varied from 4.99 to 7.32, although weakly acidic and near-neutral conditions predominated. Calcium carbonate (CaCO_3_) demonstrated the highest variability among all investigated parameters, ranging from trace amounts to 29.49%. Most soils contained less than 2% CaCO_3_, whereas isolated samples showed strong carbonate enrichment, reflecting pronounced lithological heterogeneity within the study area.

Exchangeable Ca²^+^ was the dominant base cation, ranging from 54.4 to 295.8 mmol(+)/kg and substantially exceeding Mg²^+^, Na^+^, and K^+^ concentrations. Magnesium showed moderate variability, whereas Na^+^ and K^+^ remained comparatively low in most soils.

In general, phosphorus, potassium, carbonate content, and exchangeable magnesium exhibited the strongest variability and positive skewness, whereas TOC and pH remained comparatively stable. These results demonstrate pronounced heterogeneity in nutrient distribution, carbonate content, and exchangeable cation composition across the study area. Such physicochemical heterogeneity may additionally contribute to spatial differentiation of vegetation development, nutrient cycling intensity, and ecological stability within post-mining ecosystems. The observed variability in soil physicochemical properties indicates pronounced environmental heterogeneity across the mining landscape. The predominance of silt-rich soils suggests an increased capacity for retention of fine technogenic particles and associated heavy metals because of the relatively large specific surface area of fine fractions. Consequently, granulometric composition may represent an important factor controlling contaminant accumulation and ecological risk patterns within the study area. In particular, differences in carbonate content and soil reaction suggest contrasting conditions for heavy metal retention and mobility among sampling locations.

### 3.2 Particle-size distribution and soil texture characteristics

**Figure 4:**
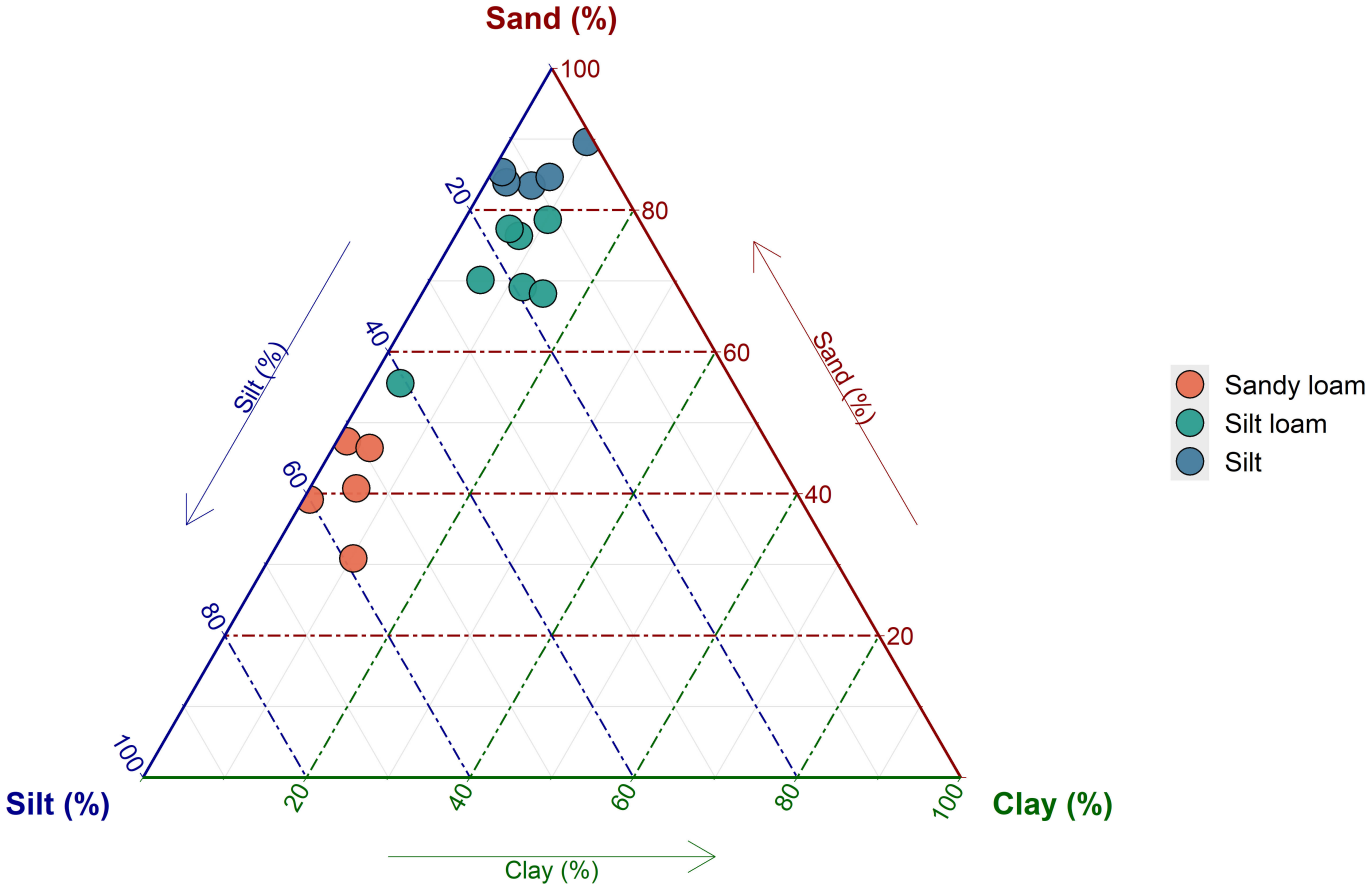
Soil texture classification of sampling sites in the Belousovka mining area based on the USDA textural triangle.

The granulometric composition of soils from the Belousovka mining area demonstrated pronounced variability in the proportions of sand, silt, and clay fractions, reflecting heterogeneous soil-forming conditions and lithological diversity (**Figure 4**). Overall, the investigated soils were predominantly characterized by elevated silt contents, whereas clay fractions remained comparatively low.

Silt was the dominant particle-size fraction in most soils, ranging from 30.87% to 89.62%. Sand contents varied between 0.93% and 60.02%, while clay fractions ranged only from 0.82% to 14.80%. Compared with the relatively stable clay distribution, both sand and silt exhibited substantially greater variability, indicating considerable spatial heterogeneity in sediment deposition and parent material composition.

According to the United States Department of Agriculture (USDA) classification, the soils were mainly represented by silt, silt loam, and sandy loam textures, with silt-dominated soils prevailing throughout the study area. Fractions smaller than 0.01 mm varied from 6.12% to 50.33%, demonstrating marked differences in the abundance of fine dispersed particles. Detailed granulometric characteristics and corresponding classifications according to both the USDA and Kachinsky systems are provided in **Supplementary Table 7**.

A clear contrast was observed between sandy and fine-textured soils. Fine-textured soils contained substantially greater proportions of fine fractions, and were associated with higher abundances of fine dispersed particles. In contrast, sandy and sandy loam soils were characterized by lower contents of fine particles and reduced proportions of fine fractions.

Overall, the texture triangle and granulometric distributions indicate that the soils of the Belousovka mining area are dominated by fine silt-rich materials with variable sand contributions and comparatively low clay contents. These textural differences were closely associated with variability in soil physicochemical characteristics across the study area. The observed variability in particle-size distribution may additionally influence water retention capacity, root penetration, vegetation establishment, and redistribution of contaminated sediments across the mining landscape.

### 3.3 Heavy metal concentrations and spatial distribution patterns

**Figure 5:**
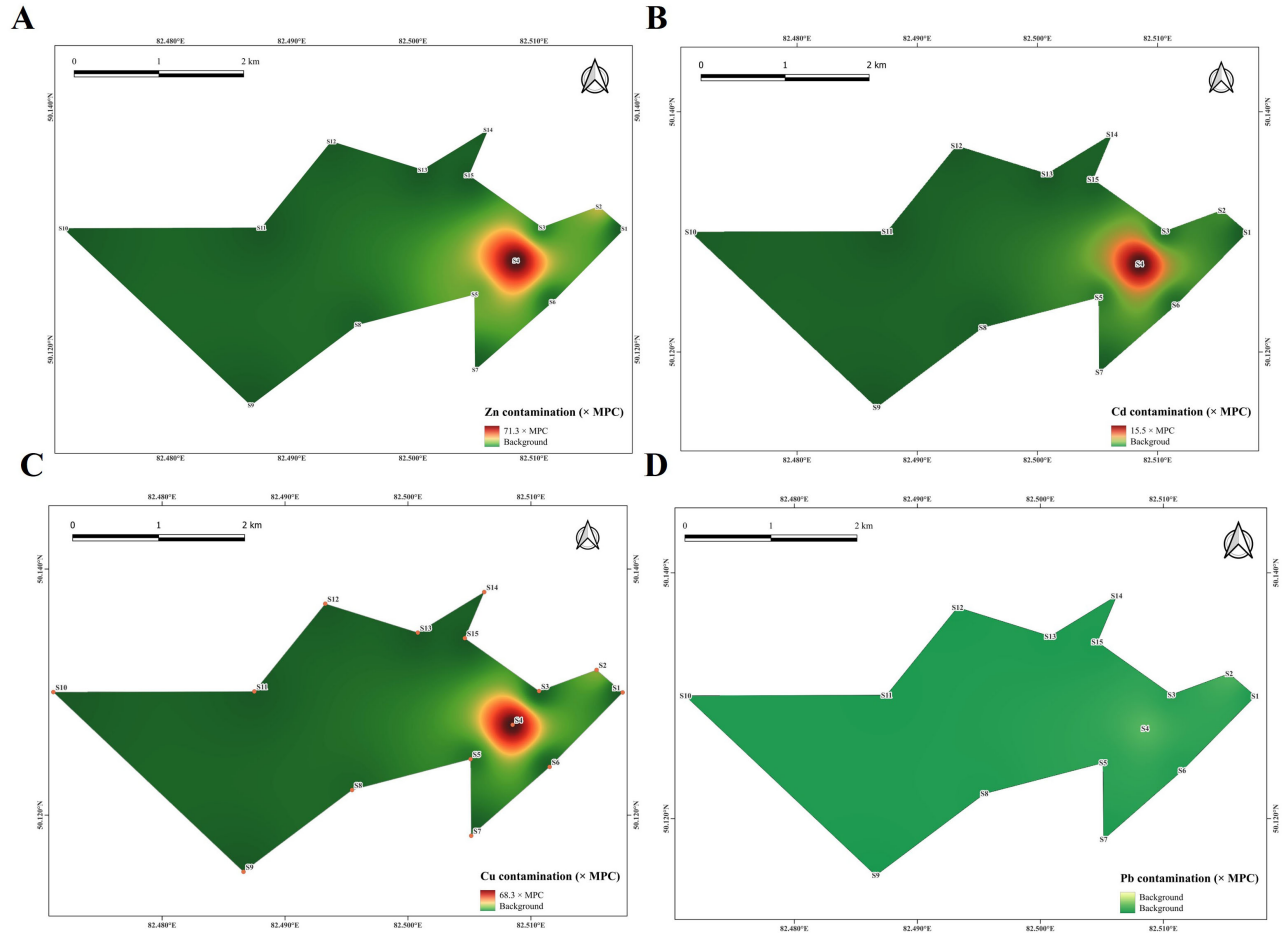
Spatial distribution of heavy metal contamination in soils of the Belousovka mining area relative to the maximum permissible concentration (MPC). Interpolated contamination maps illustrate the spatial variability of **(A)** Zn, **(B)** Cd, **(C)** Cu, and **(D)** Pb concentrations expressed as multiples of the corresponding MPC values. Warmer colors (yellow to red) indicate progressively higher contamination levels, whereas green represents background or low-contamination areas.

The investigated soils exhibited pronounced spatial heterogeneity in heavy metal accumulation, reflecting the strong influence of long-term mining activities in the Belousovka area (**Figure 5**). Among the analysed elements, Zn and Cu were the dominant contaminants, whereas Pb remained comparatively low throughout the study area.

Zn and Cu were the dominant contaminants within the investigated soils and exhibited pronounced spatial heterogeneity, reflecting the influence of historical mining activities. Cadmium occurred at lower absolute concentrations but remained environmentally significant because of its elevated toxicity, whereas Pb showed comparatively low concentrations throughout the study area. The observed contamination patterns indicate strong localized enrichment associated with former mining and ore-processing zones.

Comparison with maximum permissible concentrations (MPCs) revealed substantial anthropogenic enrichment for Zn, Cu, and Cd. Zinc exceeded MPC values by up to 71.3-fold, Cu by 68.3-fold, and Cd by 15.5-fold, whereas Pb remained below the established MPC threshold (**Supplementary Table 8**).

**Figure 6:**
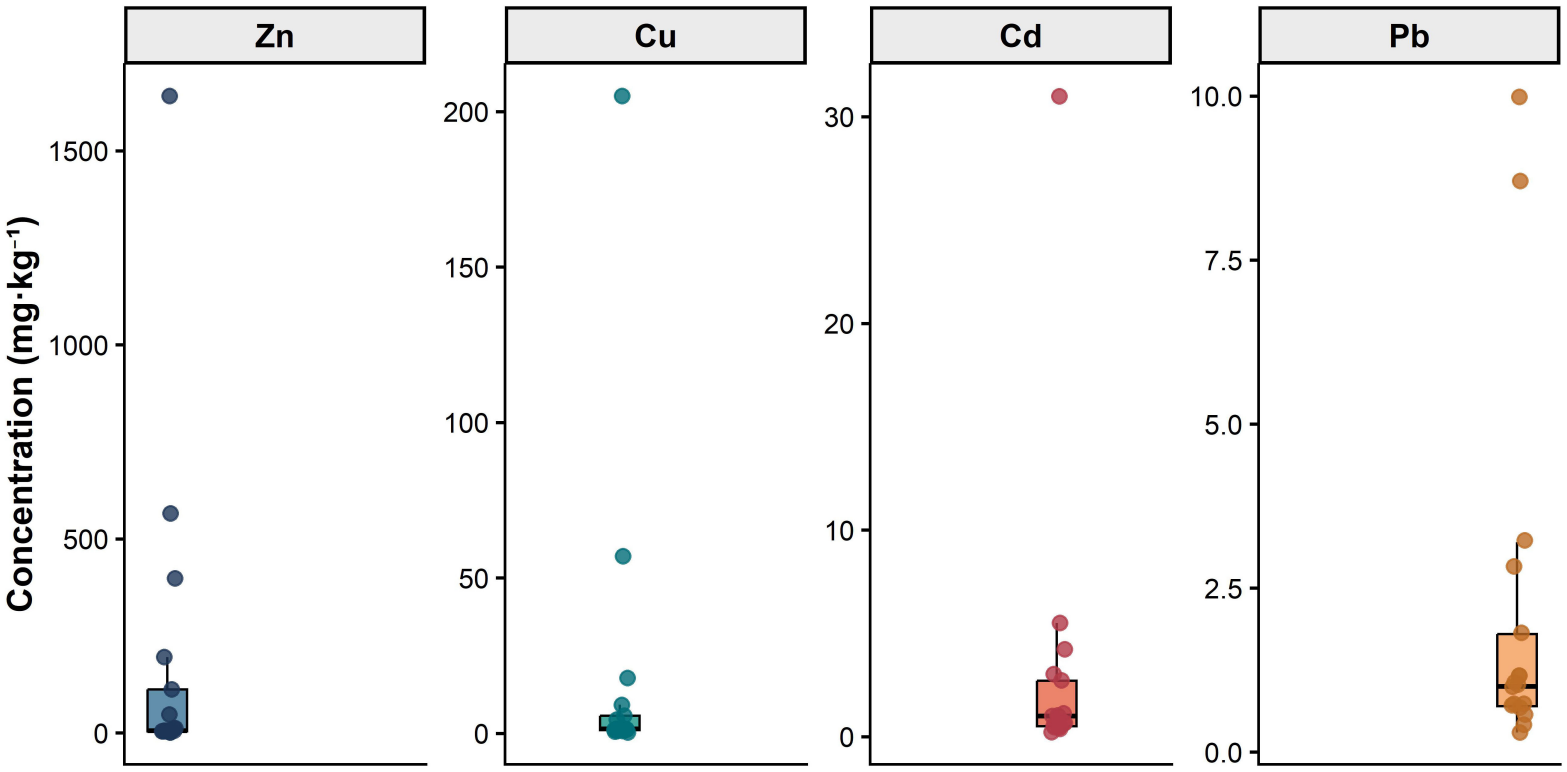
Concentrations of potentially toxic elements in soils of the Belousovka mining area.

The boxplot distributions and spatial interpolation maps identified distinct contamination hotspots for Zn, Cu, and Cd, mainly concentrated within the central-eastern part of the study area (**Figure 6**). The overlap of these enrichment zones suggests spatially associated contamination patterns.

In total, the degree of contamination followed the order Zn > Cu > Cd > Pb, confirming that Zn and Cu are the principal pollutants within the investigated soils of the Belousovka mining area.

### 3.4 Pollution assessment indices and ecological risk characterization

The integrated assessment of pollution indices revealed pronounced spatial heterogeneity in heavy metal contamination across the Belousovka mining area. The Pollution Load Index (PLI) ranged from 0.63 to 74.67, while the Potential Ecological Risk Index (RI) varied from 26.02 to 3992.46, indicating a transition from unpolluted conditions to extremely high ecological risk depending on the sampling location (**Supplementary Table 9**).

**Figure 7:**
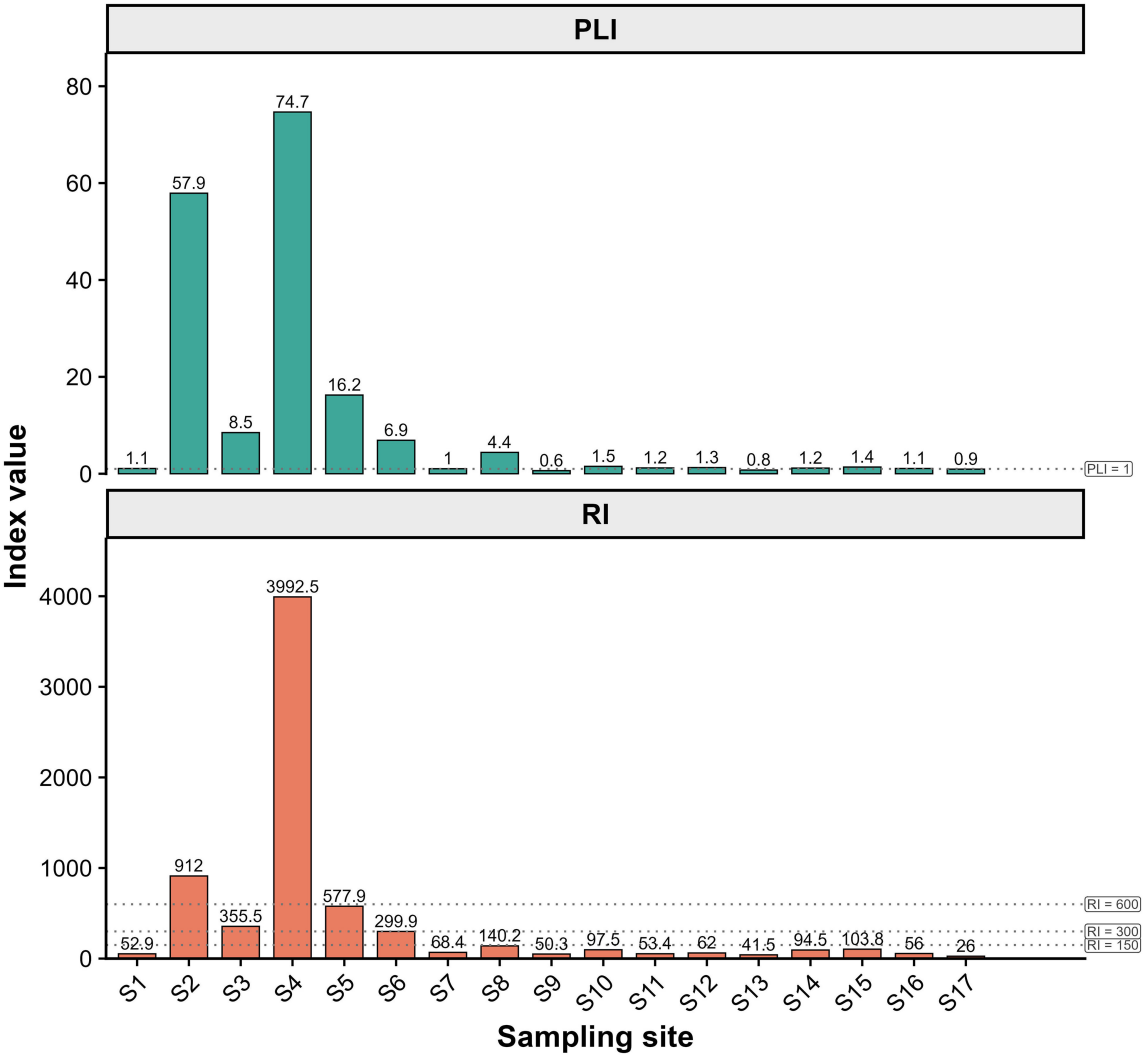
Integrated pollution load index (PLI) and ecological risk index (RI) across sampling sites in the Belousovka mining area. Dashed reference lines indicate commonly used threshold values for pollution and ecological risk classification (PLI = 1; RI = 150, 300, and 600).

The highest contamination levels were associated with sites affected by intensive mining activity, where PLI and RI values exceeded critical thresholds by several orders of magnitude (**Figure 7**). In contrast, most peripheral sites showed RI values below 150 and PLI values close to or below 1, indicating comparatively low ecological disturbance. These patterns demonstrate that ecological degradation is strongly localized near former ore-processing zones.

**Figure 8:**
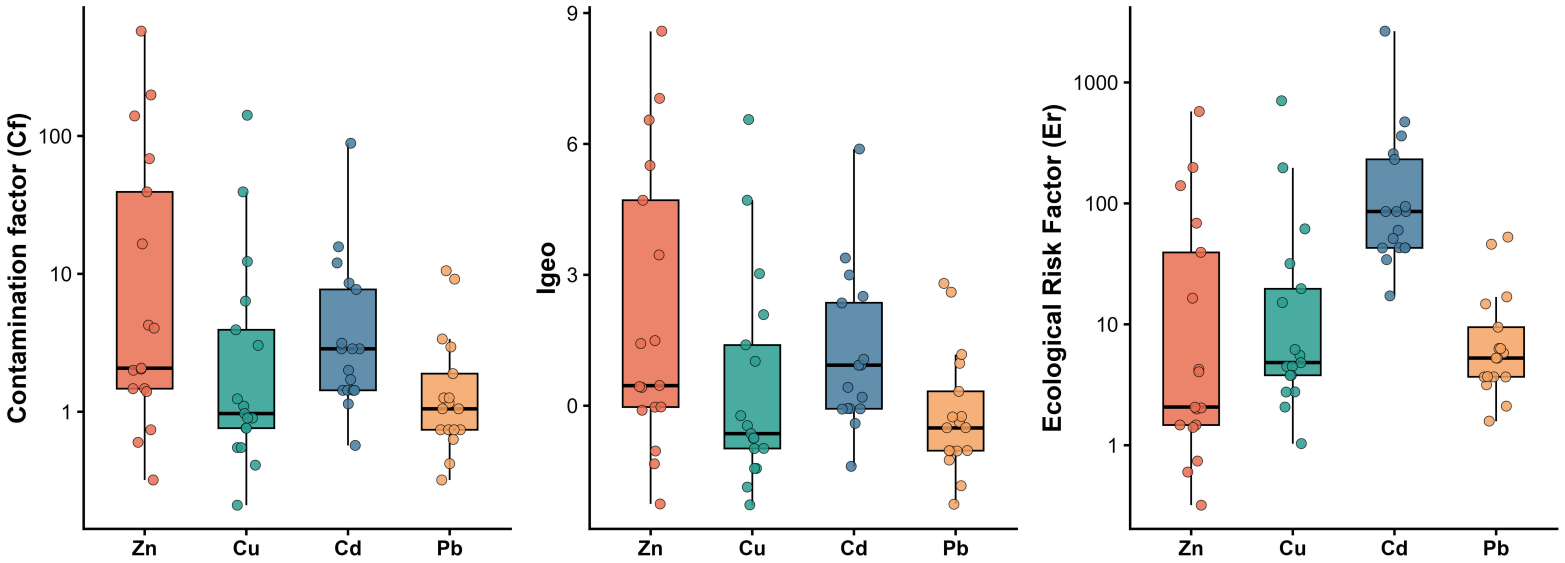
Comparative distribution of contamination factor (Cf), geoaccumulation index (Igeo), and ecological risk factor (Er) for potentially toxic elements in soils of the Belousovka mining area.

Comparative analysis of contamination factor (Cf), geoaccumulation index (Igeo), and ecological risk factor (Er) revealed substantial differences among metals (**Figure 8**). Zinc showed the highest enrichment, with Cf and Igeo values reaching 575.79 and 8.58, respectively. Copper also demonstrated strong anthropogenic accumulation, with maximum Cf and Igeo values of 141.38 and 6.56. Despite lower concentrations, Cd represented the dominant contributor to ecological risk according to Er values. The Er value for Cd reached 2657.14, substantially exceeding those of Zn, Cu, and Pb (**Supplementary Table 10**).

The boxplot distributions confirmed strong positive skewness for Zn, Cu, and particularly Cd, reflecting localized contamination hotspots superimposed on moderately polluted soils. In contrast, Pb exhibited comparatively low variability and ecological significance.

**Figure 9:**
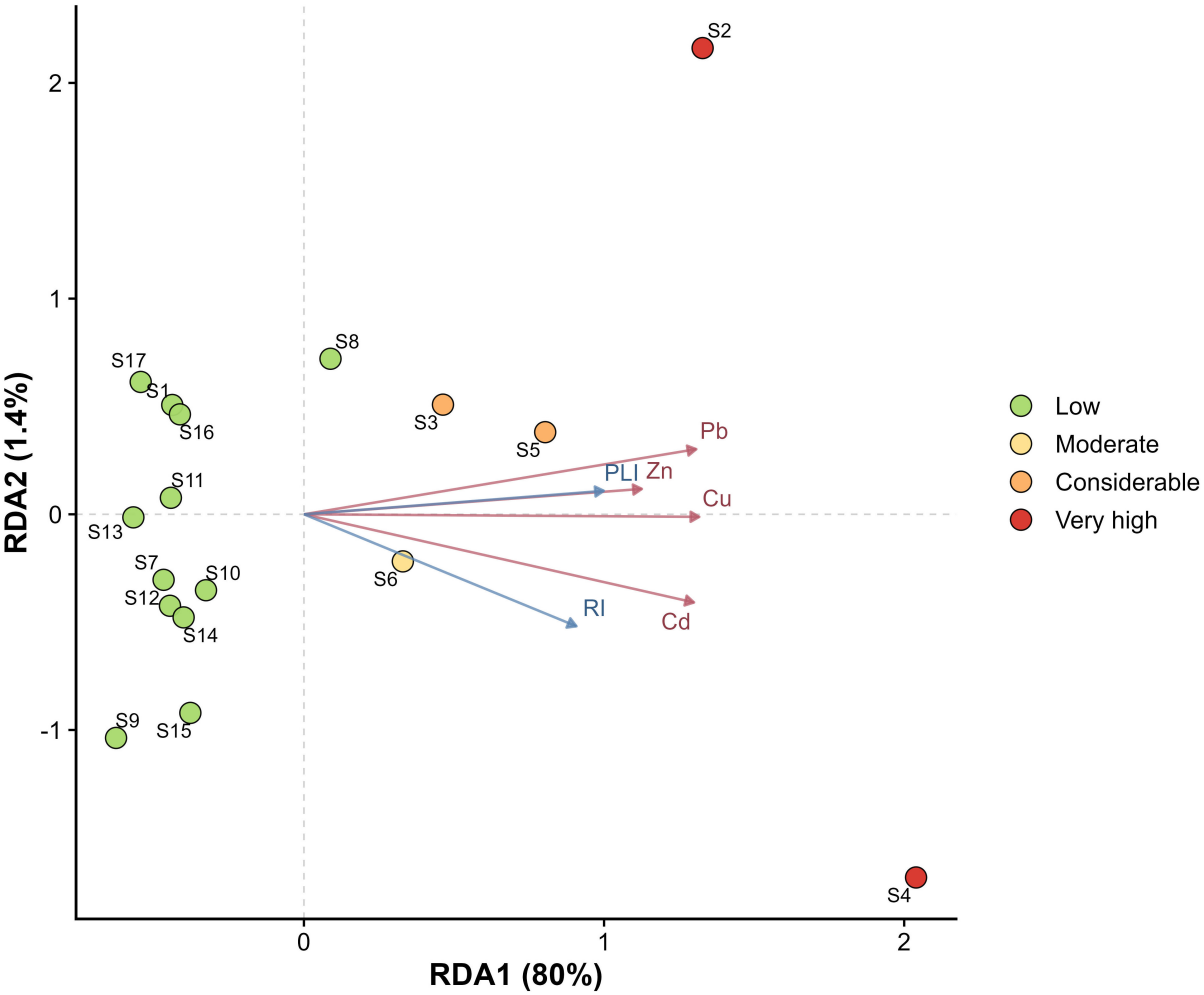
Redundancy analysis (RDA) ordination showing relationships among heavy metals, integrated pollution indices, and ecological risk groups in soils of the Belousovka mining area.

**Figure 10:**
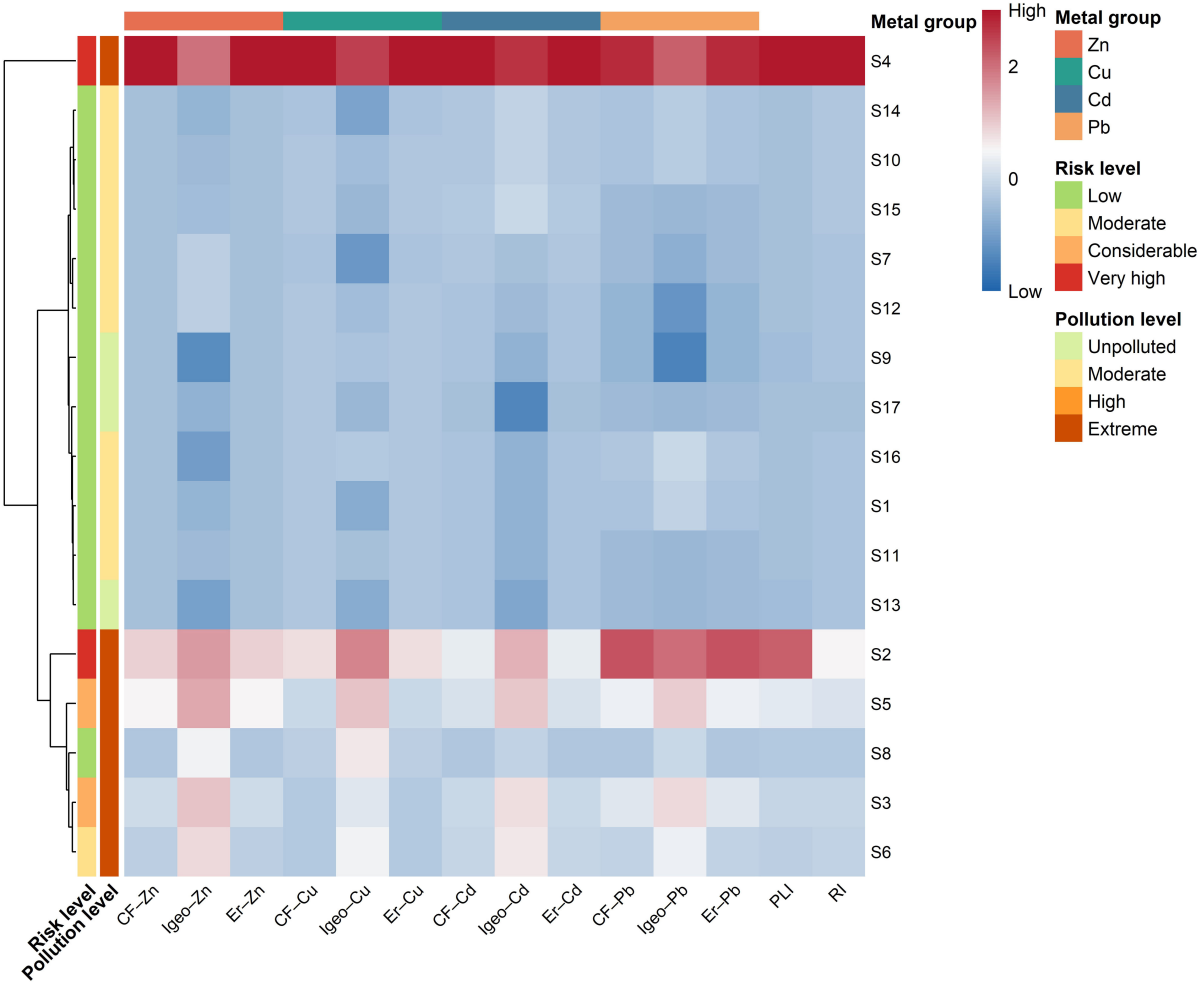
Hierarchical clustering heatmap of heavy metal pollution indices and ecological risk parameters in soils of the Belousovka mining area.

RDA and hierarchical clustering analyses supported these findings by demonstrating strong associations between Cd-related indices, RI, and PLI along the primary contamination gradient (**Figures 9** and **10**). Highly polluted sites formed distinct clusters characterized by elevated Zn-, Cu-, and Cd-associated indices, whereas low-risk soils grouped separately and displayed consistently low pollution levels.

Collectively, the results indicate that Cd is the principal ecological risk driver in the Belousovka area, while Zn and Cu are the main contributors to overall contamination intensity associated with historical mining activity.

### 3.5 Relationship between physicochemical soil properties and heavy metals

**Figure 11:**
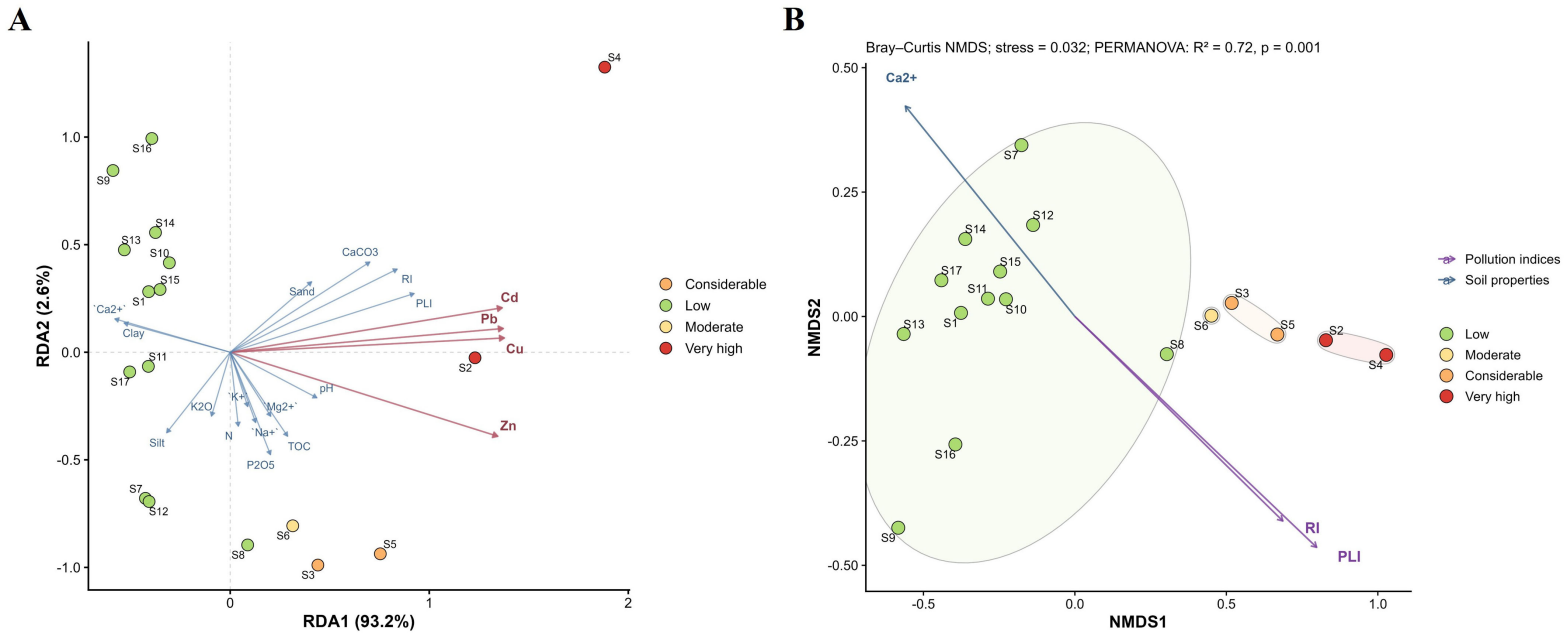
Multivariate ordination analyses of soil physicochemical properties, heavy metals, and ecological risk groups in soils of the Belousovka mining area. **(A)** Redundancy analysis (RDA). **(B)** Bray–Curtis non-metric multidimensional scaling (NMDS) with PERMANOVA statistics. Ellipses represent ecological risk categories (Low, Moderate, Considerable, and Very High) based on RI classification.

Multivariate analyses revealed strong relationships between soil physicochemical properties and heavy metal distribution in the Belousovka mining area. RDA showed that soil texture, carbonate content, salinity-related ions, and nutrient status substantially influenced the accumulation of Zn, Cu, Cd, and Pb (**Figure 11A**). The first canonical axis (RDA1) explained 93.2% of the total variance and clearly separated highly contaminated sites from low-risk soils. Samples located along the positive side of RDA1 were associated with elevated heavy metal concentrations and increased PLI and RI values, corresponding to a strong contamination gradient.

Among the investigated metals, Cd showed the strongest association with ecological risk indices, showing its strong association to environmental risk. Cd, Cu, and Pb vectors were closely aligned with highly contaminated samples, showing similar spatial distribution patterns.

NMDS analysis based on Bray–Curtis dissimilarity demonstrated clear ecological separation among contamination groups (**Figure 11B**). The ordination showed excellent goodness-of-fit (stress = 0.032), while PERMANOVA confirmed significant differences among ecological risk groups (R² = 0.72, p = 0.001). Highly contaminated soils were strongly associated with elevated PLI and RI values, whereas low-risk samples formed compact clusters characterized by lower contamination intensity.

**Figure 12:**
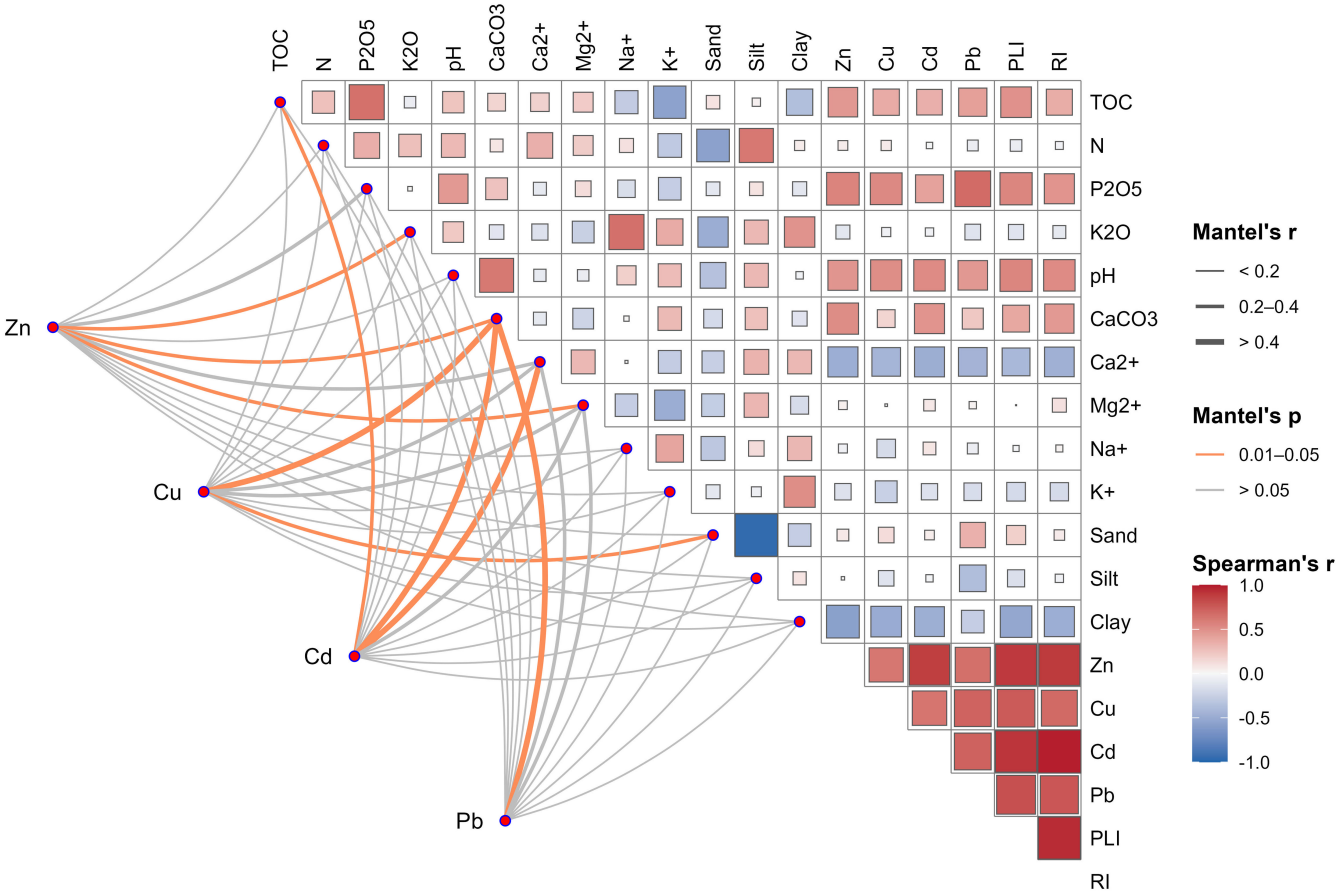
Mantel correlation network and Spearman correlation matrix illustrating relationships among soil physicochemical properties, heavy metals, and integrated pollution indices. The correlation matrix displays pairwise Spearman correlation coefficients, where square size and color intensity are proportional to the strength of the correlation. Red colors indicate positive correlations, whereas blue colors indicate negative correlations. The Mantel network links heavy metals (Zn, Cu, Cd, and Pb) with environmental variables. Line thickness is proportional to Mantel’s correlation coefficient (r), with thicker lines indicating stronger relationships. Orange links denote statistically significant Mantel correlations (0.01< p ≤ 0.05), whereas gray links indicate non-significant relationships (p > 0.05). The network highlights the environmental factors most strongly associated with heavy metal distribution and pollution patterns across the study area.

Mantel analysis identified significant positive correlations (p< 0.05) between heavy metals and CaCO_3_, pH, and sand fractions, whereas clay-rich soils showed predominantly negative relationships with Zn, Cu, Cd, Pb, PLI, and RI (**Figure 12**). Coarse-textured soils showed positive associations with heavy metal concentrations, whereas clay-rich and fine-textured soils exhibited predominantly negative relationships and lower contamination levels. Positive associations were additionally observed between Na^+^, P_2_O_5_, and several heavy metals.

Environmental fitting analysis identified Ca²^+^ as one of the main soil-related variables associated with low-risk soils, whereas PLI and RI vectors were strongly aligned with contaminated samples. The ordination additionally demonstrated a gradual transition from low-risk to very high-risk groups along the contamination gradient, demonstrating distinct separation of highly contaminated sites. This ecological separation may additionally reflect differences in vegetation cover, terrain conditions, and landscape heterogeneity across contaminated and low-risk zones.

In general, the multivariate analyses demonstrated that heavy metal distribution in Belousovka soils is controlled by both mining-related contamination and key soil physicochemical properties regulating metal mobility and ecological risk.

### 3.6 Land cover heterogeneity and terrain characteristics of the mining landscape

Land use/land cover (LULC) analysis revealed pronounced spatial heterogeneity within the Belousovka mining district, reflecting the coexistence of technogenically disturbed surfaces, semi-natural vegetation, agricultural lands, and urbanized areas (**Figure 2A**). Grasslands and tree-covered areas represented the dominant vegetation classes surrounding the former industrial infrastructure, whereas bare and sparsely vegetated surfaces were primarily concentrated near historical mining and ore-processing zones.

The observed LULC mosaic indicates substantial ecological fragmentation and uneven distribution of vegetation recovery processes across the mining landscape. Semi-natural vegetation patches and perennial grass communities were particularly widespread in peripheral and moderately disturbed areas, suggesting ongoing spontaneous succession and partial stabilization of contaminated substrates. In contrast, industrial zones characterized by limited vegetation cover may represent ecologically vulnerable areas with increased susceptibility to erosion and secondary redistribution of contaminated particles.

Topographic analysis based on the Copernicus DEM GLO-30 dataset demonstrated moderate elevation variability and heterogeneous terrain structure throughout the investigated territory (**Figure 2B**). The relief conditions suggest that terrain-controlled hydrological and erosion processes may substantially influence contaminant redistribution and localized accumulation patterns. Lower geomorphological positions potentially function as depositional zones for fine technogenic sediments, whereas elevated and exposed surfaces may experience enhanced erosion intensity and reduced retention of contaminated particles.

The combined interpretation of LULC and DEM datasets additionally indicates that vegetation structure and terrain heterogeneity may jointly regulate ecological stability, contaminant mobility, and natural recovery processes within the mining-affected ecosystem.

### 3.7 Vegetation structure and ecological characteristics of control and industrial sites

**Figure 13:**
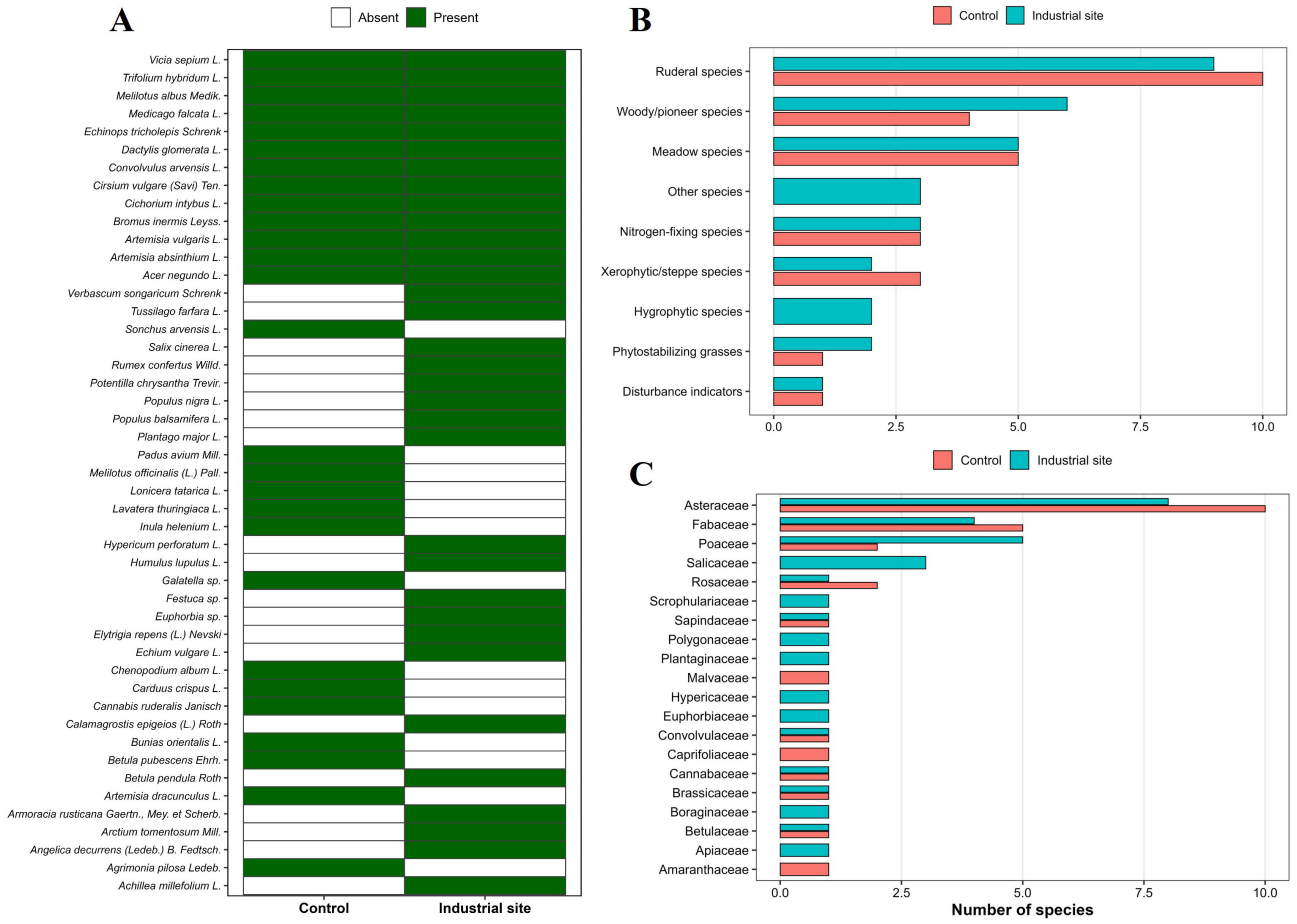
Vegetation restructuring, ecological-functional composition, and spontaneous succession patterns in mining-affected industrial and control sites: **(A)** Presence–absence heatmap of vascular plant species recorded at control and industrial sites. **(B)** Distribution of ecological-functional plant groups between control and industrial ecosystems. **(C)** Family-level floristic composition and species richness across investigated vegetation communities.

A total of 47 vascular plant species belonging to 17 families were recorded across the investigated control and industrial sites, indicating pronounced floristic heterogeneity within the mining-affected landscape (**Figure 13**). The vegetation structure was predominantly represented by *Asteraceae*, *Fabaceae*, and *Poaceae*, which together formed the major ecological and functional component of the studied phytocoenoses. The industrial site demonstrated a markedly simplified but ecologically specialized vegetation assemblage dominated by ruderal, stress-tolerant, and perennial grass species adapted to technogenically disturbed substrates.

The heatmap analysis revealed clear differentiation in species distribution between the control and industrial areas (**Figure 13A**). Several species, including *Calamagrostis epigeios*, *Elytrigia repens*, *Plantago major*, *Tussilago farfara*, *Salix cinerea*, and *Achillea millefolium*, were recorded exclusively within the industrial zone, suggesting their enhanced tolerance to disturbed soils and mining-related environmental stress. In contrast, species such as *Padus avium*, *Lonicera tatarica*, *Melilotus officinalis*, *Agrimonia pilosa*, and *Galatella* sp. occurred only at the control site and were generally associated with less disturbed meadow and semi-natural habitats. At the same time, several ecologically plastic species, including *Artemisia vulgaris*, *Dactylis glomerata*, *Vicia sepium*, *Trifolium hybridum*, and *Convolvulus arvensis*, were common to both sites, indicating broad ecological adaptability under contrasting environmental conditions.

Functional group analysis further demonstrated substantial differences in vegetation composition between the two sites (**Figure 13B**). Ruderal species represented the dominant ecological group in both areas; however, their abundance was particularly elevated within the industrial site, reflecting strong anthropogenic disturbance and active secondary succession processes. Woody and pioneer species were also more numerous in the industrial zone, where species of *Populus*, *Betula*, and *Salix* likely contribute to partial stabilization of technogenic substrates and reduction of surface erosion. In addition, phytostabilizing grasses exhibited greater representation within contaminated areas, particularly *Calamagrostis epigeios* and *Elytrigia repens*, both of which are characterized by extensive rhizomatous systems and high tolerance to heavy metal stress.

Family-level analysis additionally confirmed pronounced ecological restructuring under mining influence (**Figure 13C**). *Asteraceae* remained the dominant family at both sites, although its representation was slightly higher under control conditions. In contrast, *Poaceae* and *Salicaceae* demonstrated relatively greater importance within the industrial area, reflecting the increasing role of perennial grasses and pioneer woody species in ecological stabilization of disturbed landscapes. *Fabaceae* also maintained substantial representation across both sites, suggesting that nitrogen-fixing species may contribute to partial restoration of soil fertility and vegetation recovery under contaminated conditions.

Overall, the obtained results indicate that the industrial site is characterized by a vegetation community dominated by ruderal, perennial, and phytostabilizing species capable of persisting under elevated technogenic pressure (**Supplementary Table 11**). The observed floristic differentiation between the control and industrial areas reflects ongoing ecological filtering and adaptive vegetation restructuring within the mining-affected ecosystem.

### 3.8 Heavy metal accumulation in dominant agricultural crops

Considerable interspecific differences were observed in biomass production and heavy metal accumulation between *Helianthus annuus* L. and *Triticum aestivum* L. grown under contaminated conditions (**Table 1**). Overall, *H. annuus* demonstrated substantially greater biomass and metal accumulation capacity than *T. aestivum*, indicating higher tolerance to mining-related contamination.

**Table 1:** Heavy metal concentrations in shoots and roots of agricultural crops .

Species	Biomass (g·plant^−^¹)	Heavy metals concentrations (mg·kg^−^¹)							
		Shoot (mg·kg^−^¹, mean ± SD)				Root (mg·kg^−^¹, mean ± SD)			
		Pb	Cd	Zn	Cu	Pb	Cd	Zn	Cu
*Helianthus annuus* L.	49.01	2.7 ± 1.6 b	3.7 ± 2.2 b	55.43 ± 20.3 a	14.93 ± 9.2 b	6.18 ± 0.3 b	33.27 ± 6.8 b	572.9 ± 90.6 a	53.1 ± 33.1 b
*Triticum aestivum* L.	20.97	1.2 ± 0.3 b	0.8 ± 0.3 b	25.67 ± 9.1 a	3.73 ± 1.7 b	2.37 ± 1.7 b	11.07 ± 2.7 b	182.47 ± 83.2 a	15.8 ± 8.6 b

Data are means ± standard deviations. Different letters present significant statistical differences among different groups at the p< 0.05 level of Tukey’s HSD test, while identical letters indicate no significant difference.

The average biomass of *H. annuus* reached 49.01 g·plant^−^¹, which was more than twofold higher than that of *T. aestivum* (20.97 g·plant^−^¹). Despite exposure to elevated heavy metal concentrations, *H. annuus* maintained comparatively high biomass productivity, suggesting greater physiological adaptability under contaminated soil conditions.

Heavy metal accumulation patterns differed substantially between the two crop species. Zn was the dominant accumulated metal in both plants, followed by Cu and Cd, whereas Pb showed the lowest accumulation. Root tissues consistently contained markedly higher metal concentrations than shoots, indicating restricted translocation from belowground to aerial organs. Across all investigated metals, *Helianthus annuus* accumulated considerably greater concentrations than *Triticum aestivum*, particularly for Zn, Cu, and Cd, demonstrating stronger tolerance and accumulation capacity under mining-related contamination.

In general, the accumulation patterns followed the order Zn > Cu > Cd > Pb for both plant species, while root tissues consistently contained substantially greater heavy metal concentrations than shoots. These results indicate that root sequestration represented the dominant accumulation mechanism in both species, whereas *H. annuus* demonstrated markedly greater accumulation capacity and tolerance under contaminated environmental conditions.

### 3.9 Bioconcentration and translocation characteristics of heavy metals in dominant agricultural crops

**Figure 14:**
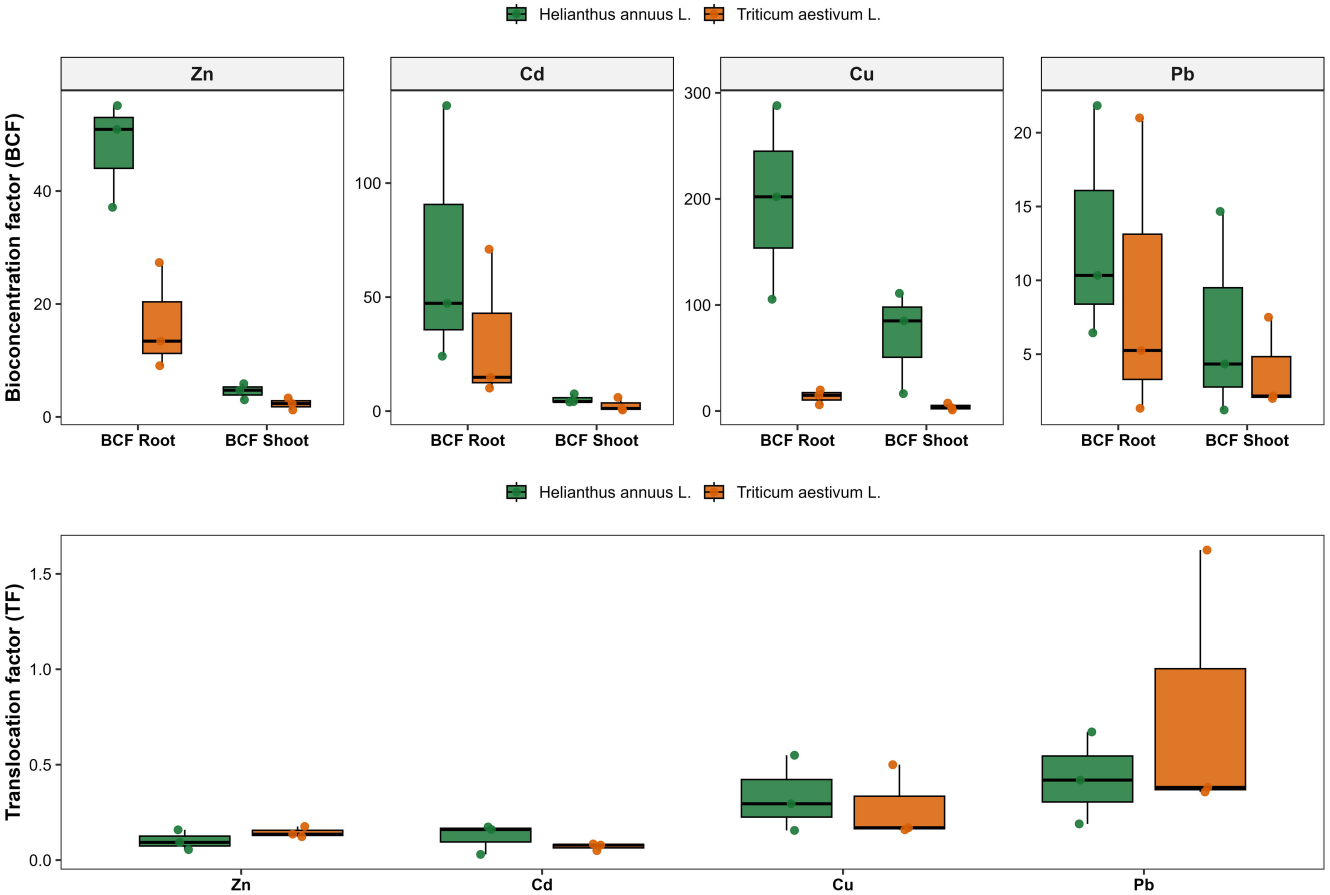
Species-specific bioconcentration and translocation responses of Zn, Cd, Cu, and Pb in plants exposed to mining-contaminated soils.

The calculated bioconcentration factor (BCF) and translocation factor (TF) values revealed pronounced differences in heavy metal uptake and internal redistribution between *Helianthus annuus* L. and *Triticum aestivum* L. (**Figure 14**). In general, *H. annuus* demonstrated substantially greater accumulation capacity for all investigated metals, particularly within root tissues, whereas *T. aestivum* showed comparatively lower uptake efficiency and reduced translocation potential.

Root BCF values consistently exceeded shoot BCF values in both species, confirming predominant retention of heavy metals within belowground tissues. *Helianthus annuus* demonstrated substantially greater accumulation efficiency than *Triticum aestivum*, particularly for Zn, Cu, and Cd. Cadmium exhibited especially strong root bioconcentration despite its comparatively lower soil concentrations, indicating high environmental mobility and bioavailability under the investigated conditions.

The TF analysis additionally confirmed limited translocation of most metals from roots to shoots. Zinc and Cd showed the lowest TF values, generally remaining below 0.2 in both plant species, indicating strong retention within root systems. Copper demonstrated slightly higher mobility, although TF values still remained below 1. In contrast, Pb exhibited the highest variability in TF, particularly in *T. aestivum*, where several values approached or exceeded unity. This suggests that Pb mobility may increase under specific local soil conditions despite its comparatively lower overall accumulation.

A clear interspecific contrast was therefore observed between the two investigated plants. *Helianthus annuus* combined high biomass production with substantially greater root accumulation capacity, particularly for Zn, Cu, and Cd, whereas *T. aestivum* maintained lower accumulation levels across both tissues. At the same time, both species demonstrated a common tendency toward root sequestration rather than efficient shoot translocation.

Overall, the results indicate that heavy metal accumulation in the investigated plants was dominated by belowground retention mechanisms. The high root BCF values combined with TF values predominantly below 1 suggest that phytostabilization rather than phytoextraction represented the principal adaptive response under mining-related contamination conditions.

## 4 Discussion

### 4.1 Physicochemical heterogeneity and geochemical controls in mining-affected soils

The physicochemical properties of the Belousovka soils indicate that the study area represents a highly heterogeneous post-mining system, where lithological variability and long-term polymetallic mining disturbance jointly control soil development. Compared with ordinary agricultural soils, mining-affected soils often show stronger spatial discontinuity in nutrient status, pH buffering, and cation composition due to mixed parent materials, spoil redistribution, and sulfide weathering processes. Similar heterogeneity has been reported in mining-affected soils, where anthropogenic disturbance altered soil nutrients, pH, and heavy metal behavior (Essandoh et al., 2021).

The TOC content in the Belousovka soils ranged from 1.83 to 5.17%, while available nitrogen varied from 33.6 to 92.4 mg·kg^−^¹. These values indicate moderate organic matter accumulation and suggest that nitrogen retention remains closely linked to soil organic carbon dynamics. Recent studies have highlighted the importance of ecological stoichiometry in regulating biogeochemical processes and elemental transport within terrestrial ecosystems. Liu et al. (2023) demonstrated that soil stoichiometric characteristics can synchronously regulate nutrient dynamics and elemental fluxes, emphasizing the critical role of soil properties in controlling the mobility and redistribution of chemical constituents. Although their study focused primarily on nitrogen and phosphorus cycling, the underlying concept provides a valuable framework for understanding how soil physicochemical conditions may also influence the retention, mobility, and environmental behavior of potentially toxic elements in mining-affected soils. This agrees with studies showing that organic matter is a key regulator of nitrogen storage, microbial activity, and recovery processes in degraded mine soils (Sahlaoui et al., 2024). In contrast, some mined soils have been reported to contain very low humus contents below 2%, especially where coarse substrates and erosion dominate, indicating that several Belousovka sites still retain comparatively better organic matter conditions (Alekseenko et al., 2022).

Available P_2_O_5_ and exchangeable K_2_O showed much stronger variability, reaching 216 and 640 mg·kg^−^¹, respectively. Such elevated values, especially at isolated sites, suggest localized lithogenic enrichment rather than uniform pedogenic accumulation. This pattern is consistent with previous studies showing that nutrient distribution in mining landscapes is often controlled by mineralogical composition, weathering intensity, and microscale redistribution. Phosphorus behavior is particularly sensitive to reactions with Fe-, Al-, and Ca-bearing minerals, which can create strong spatial contrasts in its availability.

The pH range of 4.99–7.32 indicates the coexistence of locally acidic and near-neutral geochemical environments. In polymetallic mining areas, acidic conditions may result from sulfide oxidation, whereas neutral conditions usually reflect carbonate buffering. The predominance of weakly acidic to neutral soils in Belousovka suggests that acidification is spatially limited and partly neutralized by carbonate-bearing materials. This interpretation agrees with studies demonstrating that carbonate rocks can buffer acidity and reduce the mobility of Cd, Zn, and Pb in mining environments (Lin et al., 2023).

The CaCO_3_ content ranged from trace levels to 29.49%, confirming strong lithological heterogeneity. Most samples contained low carbonate contents, whereas individual sites showed pronounced carbonate enrichment. This uneven carbonate distribution is environmentally important because CaCO_3_ regulates pH, sorption capacity, and metal immobilization. Similar studies have shown that calcite-rich soils may have strong buffering capacity and can limit the mobility of potentially toxic elements (Achour et al., 2022).

The dominance of exchangeable Ca²^+^, which reached 295.8 mmol(+)/kg, further confirms the role of carbonate and Ca-bearing minerals in controlling soil chemistry. Compared with Ca²^+^, Mg²^+^ was more variable, while Na^+^ and K^+^ remained generally low, indicating that salinization is not a dominant process in the study area. Overall, the Belousovka soils show a mosaic physicochemical structure in which carbonate-buffered microsites, localized nutrient enrichment, and organic matter-controlled nitrogen retention jointly determine soil functioning and ecological stability.

### 4.2 Granulometric heterogeneity and sedimentological controls in mining-affected soils

The granulometric composition of the Belousovka soils reflects complex sedimentological and pedogenic processes associated with polymetallic mining disturbance and lithological variability. In contrast to well-developed zonal soils, mining-affected substrates frequently exhibit heterogeneous particle-size distributions resulting from spoil redistribution, weathering of mixed parent materials, and erosion-driven sediment transport. The predominance of silt-rich materials observed in the present study is consistent with previous investigations of post-mining environments, where fine aeolian and weathered fractions commonly accumulate in depressional and low-relief areas (Abbadi and Mucsi, 2024).

The investigated soils were dominated by silt fractions, which reached up to 89.62%, whereas clay contents remained comparatively low and did not exceed 14.80%. Such texture patterns indicate that physical weathering and sediment redeposition processes play a stronger role than advanced clay mineral formation in controlling soil texture within the Belousovka area. Similar predominance of silt-rich substrates has been reported in disturbed mining landscapes characterized by mechanically fragmented parent material and weak pedogenic differentiation (Clunes et al., 2022). The relatively limited clay accumulation may additionally reflect insufficient long-term stabilization of secondary minerals under unstable post-mining conditions.

At the same time, the substantial variability in sand content, ranging from 0.93% to 60.02%, demonstrates strong spatial contrasts in depositional environments and parent material composition. Sandy loam textures identified at sites such as S2, S4, S11, and S14 likely correspond to zones affected by coarse sediment accumulation or localized spoil deposition. In contrast, highly silty soils, particularly at S7, S8, S9, and S15, indicate preferential accumulation of fine suspended material. Comparable fine-particle enrichment has been documented in mining regions where hydrological redistribution and aeolian transport promote selective deposition of silt fractions (Gebre et al., 2023).

The distribution of fine dispersed particles (<0.01 mm), which reached 50.33% in some samples, is particularly important because fine fractions strongly influence sorption capacity, aggregate stability, water retention, and trace metal accumulation. Numerous studies have demonstrated that silt- and clay-rich soils possess greater capacities for immobilization of potentially toxic elements due to their higher specific surface area and abundance of reactive mineral phases (Mensah and Amoakwah, 2024). Consequently, the predominance of fine-textured materials in the Belousovka area may significantly affect the spatial distribution and retention of heavy metals.

The contrast between coarse-textured and fine-textured soils also has important ecological implications. Sandy soils generally exhibit lower nutrient retention and weaker structural stability because of reduced contents of fine dispersed particles and organic-mineral complexes. In contrast, fine-textured soils characterized by elevated proportions of silt and clay fractions. This relationship likely explains part of the variability previously observed in physicochemical parameters, particularly nutrient availability and exchangeable cation composition.

The USDA classification indicates that silt, silt loam, and sandy loam soils dominate throughout the study area, reflecting substantial granulometric heterogeneity and variable capacities for water retention and contaminant immobilization. Such granulometric diversity suggests that soil formation in the Belousovka mining district proceeds under highly heterogeneous sedimentological conditions controlled by mixed lithology and long-term technogenic redistribution. Overall, the observed textural variability represents a key factor governing nutrient dynamics, water retention, and contaminant mobility within the investigated post-mining ecosystem.

### 4.3 Spatial patterns and geochemical controls of heavy metal accumulation in mining-affected soils

The distribution of heavy metals in the Belousovka soils clearly reflects the long-term influence of polymetallic mining activities and associated geochemical redistribution processes. The predominance of Zn and Cu contamination, together with comparatively low Pb concentrations, indicates that the investigated soils preserve a distinct metallogenic signature characteristic of Zn–Cu ore mineralization. Similar contamination profiles have been reported in polymetallic mining districts where sulfide ore extraction and waste deposition resulted in selective enrichment of Zn, Cu, and Cd within surrounding soils (Sun et al., 2018).

Among the investigated elements, Zn exhibited the strongest enrichment and spatial variability, reaching 1641 mg·kg^−^¹ at site S4. Such concentrations substantially exceed typical regional background levels reported for uncontaminated soils and indicate intense localized anthropogenic accumulation. The observed Zn and Cu concentrations also exceed commonly reported international soil quality guideline values for agricultural and residential soils, further confirming the severe degree of contamination within localized sectors of the Belousovka mining area. Such exceedances indicate that historical mining activities have resulted in metal accumulation levels that may pose long-term ecological constraints for sustainable land use and ecosystem functioning. Copper showed a similar spatial pattern, with concentrations up to 205 mg·kg^−^¹, whereas Cd reached 31 mg·kg^−^¹ despite considerably lower absolute concentrations. The overlap of Zn-, Cu-, and Cd-enriched zones within the central-eastern sector of the study area strongly suggests a common contamination source associated with historical ore extraction, processing activities, and dispersion of mining wastes. This interpretation is further supported by Qian et al. (2025), who demonstrated that heavy metals can be continuously released from industrial and mining-related wastes through geochemically driven leaching processes. Their findings indicate that weathering and chemical transformation of metal-bearing materials may act as long-term sources of contaminant release, sustaining elevated concentrations in surrounding environments. Although the study was performed under controlled experimental conditions, the identified release mechanisms provide a useful framework for understanding the persistence of Zn, Cu, and Cd contamination and the development of localized enrichment hotspots observed in the Belousovka mining district. Comparable hotspot-type distributions have been widely documented in abandoned mining regions, where atmospheric deposition, runoff transport, and tailing weathering produce highly localized contamination anomalies (Ni et al., 2023). Mining-related contamination is also closely linked to broader landscape and hydrological processes. Feng et al. (2025) demonstrated that long-term mining activities may substantially alter surface water dynamics, hydrological connectivity, and sediment redistribution patterns within mining landscapes. Such processes can influence the transport and accumulation of contaminated particles, thereby contributing to the spatial heterogeneity of heavy metal distribution observed in post-mining environments. Consequently, the contamination patterns identified in the Belousovka area likely reflect the combined influence of geochemical, geomorphological, and hydrological controls operating across multiple spatial scales.

The magnitude of Zn enrichment observed in the Belousovka soils is particularly significant. Concentrations exceeding permissible limits by more than 70-fold indicate severe technogenic disturbance and prolonged accumulation of Zn-bearing particles within the soil system. Similar Zn anomalies have been described in Pb–Zn mining areas of China, Eastern Europe, and Central Asia, where weathering of sphalerite-rich tailings generated persistent contamination of adjacent soils (Guo et al., 2022). Zinc mobility in such environments is strongly influenced by pH, carbonate content, and fine particle abundance, which likely explains the pronounced spatial heterogeneity observed across the study area. In addition, Zn is commonly associated with sphalerite (ZnS), one of the principal ore minerals occurring within polymetallic deposits of Eastern Kazakhstan. Progressive weathering of sulfide-bearing wastes and tailings may therefore provide a long-term source of Zn release, contributing to persistent soil enrichment and the formation of localized contamination hotspots.

Cadmium concentrations, although lower in absolute magnitude, are environmentally important because Cd exhibits substantially higher toxicity and mobility compared with many other trace metals. The elevated Cd levels recorded at several sampling sites indicate potential ecological risk even under relatively neutral soil conditions. Numerous studies have emphasized that Cd can remain highly bioavailable in mining soils due to weak sorption stability and competitive interactions with Zn and Ca (Padoan et al., 2020). Consequently, the coexistence of elevated Zn and Cd concentrations may substantially increase environmental vulnerability within contaminated microsites. The elevated ecological significance of Cd is additionally related to its weak sorption relative to Pb and its ability to compete with Ca²^+^ and Zn²^+^ for adsorption sites within the soil matrix. As a result, Cd may remain more mobile and bioavailable than many other trace metals, facilitating its transfer into biological systems.

In contrast, Pb concentrations remained comparatively low and demonstrated weak spatial differentiation throughout the study area. This behavior differs from many Pb–Zn mining districts, where lead commonly represents one of the dominant contaminants. The relatively limited Pb accumulation in the Belousovka soils may reflect lower Pb abundance within local ore assemblages or stronger immobilization through adsorption and carbonate precipitation processes. Previous investigations have shown that Pb generally exhibits lower mobility than Zn and Cd due to its strong affinity for organic matter, carbonates, and Fe-Mn oxides (Kicińska et al., 2019).

The observed contamination patterns additionally demonstrate the important role of soil physicochemical and granulometric properties in controlling trace metal redistribution. Fine-textured soils enriched in silt and fine dispersed particles may enhance metal retention because of their greater sorption capacity and larger reactive surface area. Therefore, the spatial overlap between fine-grained substrates and contamination hotspots likely contributes to stabilization of heavy metals within specific sectors of the mining landscape (Lv et al., 2022).

Overall, the obtained results indicate that the Belousovka soils remain under strong technogenic geochemical influence, with Zn and Cu representing the principal contaminants and Cd constituting an additional ecotoxicological concern. The pronounced spatial heterogeneity of metal accumulation highlights the importance of localized lithological and sedimentological controls in regulating contaminant mobility and ecological risk within post-mining ecosystems.

### 4.4 Integrated pollution indices and ecological risk differentiation in mining-affected soils

The integrated pollution assessment demonstrates that ecological degradation in the Belousovka mining area is not spatially uniform but strongly concentrated in several contamination hotspots. This pattern is consistent with the conceptual basis of the Pollution Load Index and Potential Ecological Risk Index, which are widely used to distinguish cumulative contamination intensity from metal-specific toxicological risk. The PLI was originally proposed as an integrated indicator of overall heavy metal pollution, whereas RI combines contamination level with toxic-response coefficients, making it particularly sensitive to highly toxic elements such as Cd (Hakanson, 1980).

In the present study, PLI values ranged from 0.63 to 74.67, while RI varied from 26.02 to 3992.46, indicating a sharp transition from low-disturbance peripheral soils to extremely contaminated sites near former mining-related zones. The most critical site was S4, where PLI reached 74.67 and RI reached 3992.46, confirming extreme cumulative pollution and very high ecological risk. Similar hotspot-driven patterns have been reported in mining-affected soils, where local ore-processing residues and tailings generate abrupt increases in pollution indices rather than gradual regional contamination (Wang et al., 2025).

The metal-specific indices clarify the mechanisms behind this risk structure. Zn showed the strongest contamination intensity, with maximum Cf and Igeo values of 575.79 and 8.58, respectively, followed by Cu with Cf = 141.38 and Igeo = 6.56. According to the geoaccumulation concept introduced by Müller, such Igeo values correspond to extremely contaminated conditions (Müller, 1979). However, ecological risk was not controlled primarily by the most abundant metals. Instead, Cd was the dominant risk driver, with Er reaching 2657.14, far exceeding Zn, Cu, and Pb. This agrees with previous studies showing that Cd often contributes disproportionately to ecological risk because of its high toxic-response coefficient, mobility, and biological availability (Jiang et al., 2014).

The contrast between contamination intensity and ecological risk is therefore central to interpreting the Belousovka soils. Zn and Cu define the magnitude of technogenic enrichment, whereas Cd determines the ecological severity of contamination. This distinction is important because reliance only on total metal concentrations may underestimate ecological risk in areas where Cd concentrations are lower than Zn or Cu but toxicologically more significant. Similar conclusions were reported in studies applying ecological risk indices to contaminated soils and sediments, where Cd frequently dominated RI despite not being the most abundant element (Ogunkunle and Fatoba, 2013). Despite their widespread application, the Potential Ecological Risk Index (RI) and individual ecological risk factor (E_r_) possess several limitations when applied to terrestrial ecosystems. These indices are based primarily on total metal concentrations and predefined toxic-response coefficients and therefore do not explicitly account for metal speciation, bioavailability, soil physicochemical properties, biological uptake, or site-specific ecological sensitivity. Consequently, RI and E_r_ should be interpreted as screening-level indicators of potential ecological hazard rather than direct measures of ecological effects. For this reason, the present study combined pollution indices with soil physicochemical characterization, multivariate analyses, vegetation assessment, and plant accumulation data to provide a more comprehensive evaluation of environmental risk. An additional limitation should be acknowledged regarding the background concentrations used for pollution index calculations. Because regional geochemical baseline values are currently unavailable for the Belousovka mining district, local reference sites (S16 and S17) were used as surrogate background locations. From a practical risk-management perspective, the wide range of PLI (0.63–74.67) and RI (26.02–3992.46) values reflects substantial spatial variability in contamination severity across the mining landscape. Sites characterized by low PLI and RI values may require only routine environmental monitoring, whereas locations exhibiting extremely high contamination and ecological risk indices should be considered priority targets for remediation planning, land-use management, and long-term environmental surveillance. The exceptionally high RI values, primarily associated with Cd contamination, identify ecological hotspots where mitigation measures should be prioritized to reduce potential environmental impacts.

The clustering and RDA patterns further support the presence of two contrasting soil groups: highly impacted sites associated with Zn–Cu–Cd enrichment and low-risk peripheral sites characterized by weak pollution signals. Overall, the Belousovka area represents a strongly heterogeneous post-mining system in which cumulative pollution is mainly driven by Zn and Cu, while ecological risk is controlled by Cd toxicity. These findings highlight the need for site-specific remediation prioritization, with particular attention to Cd-enriched hotspots rather than only areas with the highest total metal loads.

### 4.5 Soil property controls on heavy metal distribution and ecological risk gradients

The multivariate structure of the Belousovka dataset indicates that heavy metal accumulation is controlled not only by proximity to mining sources but also by soil properties regulating retention, mobility, and geochemical partitioning. This interpretation is supported by the RDA results, where the first axis explained 93.2% of the variance and separated highly contaminated sites from low-risk soils. The exceptionally high proportion of variance explained by the first RDA axis most likely reflects the presence of a dominant contamination gradient rather than excessive multicollinearity among explanatory variables. Prior to ordination, multicollinearity was evaluated using variance inflation factors (VIF), and variables with VIF values > 10 were excluded from the analysis. Therefore, the strong separation observed along RDA1 indicates that heavy metal contamination and associated ecological risk were structured primarily by a common technogenic gradient across the study area. Such a strong gradient is typical of mining-affected landscapes, where local contamination sources interact with soil texture, pH, carbonate content, and exchangeable cations to determine metal distribution. Similar mechanisms have been described in contaminated soils, where pH, organic matter, clay minerals, and carbonates are key regulators of metal solubility and bioavailability (Wuana and Okieimen, 2011). This interpretation is further supported by Wang et al. (2024), who demonstrated that changes in soil chemical conditions can strongly influence trace element mobilization and immobilization processes by altering geochemical partitioning and element–soil interactions. Although their study focused on arsenic in flooded agricultural soils, the underlying mechanisms emphasize the critical role of soil physicochemical properties in controlling contaminant mobility, retention, and environmental risk, which is consistent with the patterns observed for Zn, Cu, and Cd in the Belousovka mining soils.

The alignment of Cd, Cu, Pb, PLI, and RI vectors with highly contaminated samples demonstrates that ecological risk in Belousovka is strongly structured by a common contamination gradient. This is consistent with previous studies showing that multivariate ordination effectively distinguishes polluted and less polluted soil groups in mining regions. In the present study, this separation was particularly clear: NMDS produced a very low stress value of 0.032, while PERMANOVA confirmed significant differences among risk groups (R² = 0.72, p = 0.001). These values indicate a robust ecological separation between peripheral low-risk soils and highly impacted sites.

The positive relationships between heavy metals and CaCO_3_, pH, and sand fractions suggest that contamination hotspots are partly associated with carbonate-bearing and coarse-textured substrates. Although carbonates can immobilize metals by precipitation and adsorption, their association with contamination in Belousovka likely reflects lithological control and the deposition of mineralized mining material rather than enhanced mobility. Similar findings have been reported for mining soils where carbonate-rich substrates buffer acidity but may also coincide with ore-derived metal accumulation (Yang et al., 2018). Carbonate-rich substrates may therefore play a dual ecological role by simultaneously acting as repositories of ore-derived contaminants and geochemical barriers that reduce dissolved metal concentrations through precipitation and adsorption processes.

The negative association of clay-rich soils with Zn, Cu, Cd, Pb, PLI, and RI is also noteworthy. In many soils, clay fractions enhance metal retention because of their high surface area and cation-exchange capacity; however, in Belousovka, lower contamination in clay-rich samples likely reflects their location outside the main technogenic deposition zone. This emphasizes that spatial source intensity may override the expected sorption effect of fine particles in strongly heterogeneous mining landscapes.

The NMDS environmental fitting further showed that Ca²^+^ was associated with low-risk soils, whereas PLI and RI were aligned with contaminated samples. This suggests that Ca-rich conditions may contribute to buffering in peripheral soils, while risk escalation is primarily driven by Zn–Cu–Cd enrichment near former mining zones. From a conceptual perspective, the spatial distribution of heavy metals and associated ecological risk in the Belousovka mining district can be explained by a coupled source–property control framework operating across multiple environmental scales. Within this framework, historical mining activities, ore-processing residues, tailings, and the progressive weathering of metal-bearing materials constitute the primary contamination sources that determine the magnitude, composition, and spatial intensity of metal inputs. However, the environmental fate of these contaminants is not governed solely by source strength. Instead, metal mobility, retention, redistribution, and ecological availability are further regulated by a suite of interacting soil and landscape factors, including texture, pH, carbonate content, organic matter, exchangeable cations, and terrain-driven transport processes. These environmental controls influence sorption–desorption equilibria, precipitation and dissolution reactions, metal complexation, and the accumulation of contaminated fine particles within depositional environments. Consequently, the observed contamination hotspots and ecological risk gradients reflect the combined influence of contaminant loading and site-specific geochemical buffering capacity. In this context, ecological risk emerges as an integrated outcome of source intensity, metal mobility, and environmental stabilization processes rather than a direct function of total metal concentrations alone. Furthermore, these source–property interactions indirectly influence vegetation establishment, plant metal uptake, and phytostabilization efficiency by modifying metal bioavailability and the physicochemical conditions governing plant growth. Overall, the Belousovka soils demonstrate a coupled source–property control system, where mining-derived contamination defines the main risk gradient and soil physicochemical properties modulate metal stabilization and ecological impact.

### 4.6 Ecological implications of land cover heterogeneity and terrain-controlled contaminant redistribution

The obtained results indicate that ecological functioning within the Belousovka mining landscape is controlled not only by the intensity of heavy metal contamination but also by the spatial heterogeneity of land cover and topographic conditions (Gataulina et al., 2025). The coexistence of industrially disturbed surfaces, agricultural lands, grasslands, wetlands, and semi-natural vegetation suggests the formation of a fragmented post-mining ecological mosaic characterized by strong contrasts in soil stability, contaminant mobility, and habitat quality. Similar landscape-scale heterogeneity has been reported in post-industrial ecosystems where vegetation structure and geomorphological variability substantially regulate the redistribution of contaminated sediments and ecological recovery processes (Rahmonov et al., 2021).

The predominance of grassland and tree-covered patches surrounding contaminated zones may play an important stabilizing role within the investigated territory. Vegetation cover can significantly reduce erosion intensity, increase soil aggregation, enhance organic matter accumulation, and limit aeolian transport of fine technogenic particles enriched with heavy metals. In addition, root systems may contribute to mechanical stabilization of contaminated substrates and reduce hydrological redistribution of polluted sediments during seasonal runoff events. Comparable ecological functions of spontaneous vegetation and semi-natural plant communities have been widely documented in mining-affected environments undergoing natural succession and phytostabilization processes (Álvarez-Rogel et al., 2022).

In contrast, bare and sparsely vegetated surfaces identified near former industrial and ore-processing zones likely represent ecologically vulnerable hotspots characterized by enhanced erosion susceptibility and reduced biological resilience. The absence of stable vegetation cover may substantially increase the mobility of fine contaminated particles, particularly under strongly continental climatic conditions associated with seasonal runoff and wind redistribution (Yu et al., 2025). Such disturbed substrates commonly exhibit limited nutrient retention, reduced microbial activity, and weak structural stability, which together suppress vegetation recovery and delay ecological succession. Similar degradation patterns have been observed in polymetallic mining districts where unvegetated surfaces function as secondary sources of contaminant dispersion (Goulier et al., 2020).

Topographic heterogeneity additionally appears to exert strong control over the spatial redistribution of contaminants across the study area. DEM-based analysis suggests that lower geomorphological positions may preferentially accumulate fine-grained technogenic sediments transported by surface runoff, thereby forming localized contamination hotspots (Hošek et al., 2024). This interpretation is supported by the elevated heavy metal concentrations and ecological risk values observed within several low-relief depositional environments. Conversely, elevated and exposed slopes are more likely to experience intensified erosion, periodic removal of fine fractions, and reduced accumulation of organic matter and clay particles. Terrain-controlled redistribution processes therefore contribute not only to geochemical differentiation but also to ecological fragmentation within the post-mining landscape. Similar relationships between relief conditions, sediment transport, and heavy metal accumulation have been reported in mountainous mining regions affected by long-term technogenic disturbance (Sanad et al., 2025).

Collectively, these findings indicate that vegetation structure, land cover heterogeneity, and relief-driven redistribution processes jointly regulate ecosystem stability and potential biodiversity recovery within the Belousovka mining district. The observed ecological mosaic demonstrates that post-mining environments should be interpreted as spatially dynamic systems where contaminant mobility, soil development, and vegetation succession are strongly interconnected. Consequently, future remediation and monitoring strategies should integrate not only geochemical indicators but also landscape structure, vegetation recovery dynamics, and terrain-controlled ecological processes in order to improve long-term ecosystem rehabilitation and environmental sustainability.

### 4.7 Vegetation adaptation and ecological stabilization under mining-related disturbance

The pronounced differences in vegetation composition between the control and industrial sites indicate that long-term mining activity has substantially transformed the ecological structure and successional dynamics of the studied landscape. The industrial site was dominated by ruderal, perennial, and stress-tolerant species, whereas the control area retained a greater proportion of meadow and semi-natural vegetation. These results are consistent with previously conducted studies in mining-affected ecosystems, where technogenic disturbance and heavy metal contamination were identified as the principal ecological filters controlling floristic composition and vegetation succession (Chen et al., 2019).

The predominance of *Asteraceae* and *Poaceae* within the industrial site likely reflects their high ecological plasticity, efficient reproductive strategies, and tolerance to nutrient imbalance and heavy metal stress. These characteristics enable rapid colonization of disturbed substrates and promote persistence under long-term technogenic pressure. Their ecological success is further associated with extensive root systems and physiological mechanisms that reduce metal toxicity through compartmentalization and stress-tolerance responses. As a result, species belonging to these families frequently dominate early and intermediate stages of succession in disturbed mining landscapes. These adaptive traits explain why ruderal herbs and perennial grasses frequently dominate contaminated industrial and post-mining ecosystems undergoing natural succession. In particular, the widespread occurrence of *Artemisia vulgaris*, *Cirsium vulgare*, *Convolvulus arvensis*, and *Plantago major* at the industrial site is consistent with vegetation patterns previously observed in abandoned polymetallic mining regions of Eastern Europe and Central Asia (Rahmonov et al., 2021). Although no obligate heavy-metal hyperaccumulator species were identified during the survey, several dominant taxa recorded at the industrial site, including *Artemisia vulgaris*, *Plantago major*, *Calamagrostis epigeios*, *Elytrigia repens*, and *Cirsium vulgare*, have been reported as metal-tolerant species capable of colonizing contaminated substrates and persisting under elevated heavy metal concentrations. Their ecological success is associated with adaptive physiological responses such as metal sequestration in roots, compartmentalization within tissues, stress-tolerance mechanisms, and extensive root development. These traits may contribute to spontaneous phytostabilization processes and facilitate long-term stabilization of contaminated soils in mining-affected environments.

The elevated representation of perennial grasses within the industrial area further suggests ongoing ecological stabilization of technogenic substrates. The dominance of *Calamagrostis epigeios*, *Dactylis glomerata*, and *Elytrigia repens* suggests that rhizomatous growth strategies provide a substantial competitive advantage under contaminated conditions. Extensive belowground biomass enhances substrate stabilization, improves water retention, and facilitates recolonization of disturbed soils following mining activities. Similar patterns have been reported during intermediate stages of post-mining succession (Paniagua-López et al., 2024). The competitive advantage of this species is also linked to its extensive rhizome network and high regenerative capacity, which facilitate long-term persistence on unstable substrates and enhance physical stabilization of contaminated soils. The exclusive occurrence of this species within the industrial site in the present study therefore likely reflects active spontaneous phytostabilization processes under persistent technogenic pressure.

The occurrence of woody pioneer species indicates ongoing ecological succession and progressive structural development of vegetation communities. Through litter accumulation, root penetration, and microhabitat formation, pioneer trees and shrubs may accelerate soil development and facilitate the establishment of less stress-tolerant species. Comparable successional trajectories have been reported in other mining-affected landscapes undergoing spontaneous revegetation (Mendez et al., 2007). Beyond their role as pioneer colonizers, woody species may accelerate ecosystem recovery by increasing litter inputs, promoting nutrient cycling, improving soil structure, and creating microhabitats that facilitate establishment of less stress-tolerant species during later successional stages.

In contrast, the control site supported a larger proportion of meadow-associated and less disturbance-tolerant species such as *Agrimonia pilosa*, *Padus avium*, *Lonicera tatarica*, and *Melilotus officinalis*. These findings are consistent with earlier ecological studies showing that semi-natural meadow species generally decline under elevated anthropogenic disturbance and heavy metal contamination. At the same time, several ecologically plastic taxa, including *Artemisia vulgaris*, *Dactylis glomerata*, *Vicia sepium*, and *Trifolium hybridum*, occurred at both sites, indicating broad adaptive capacity under contrasting environmental conditions.

The relatively high representation of *Fabaceae* suggests an important role of biological nitrogen fixation in ecosystem recovery. Symbiotic associations with rhizosphere microorganisms may improve soil fertility, enhance nutrient cycling, and facilitate establishment of subsequent plant communities in degraded mining substrates. Similar ecological functions of legumes have been documented in restoration studies of post-industrial ecosystems (Gornish and Ambrozio dos Santos, 2016). The ecological importance of legumes is further enhanced by their symbiotic associations with nitrogen-fixing microorganisms, which may increase soil fertility and accelerate successional development in degraded mining landscapes where nutrient availability is often limited.

Overall, the obtained vegetation patterns are in good agreement with previously reported ecological succession models for mining-affected ecosystems. The dominance of ruderal herbs, perennial grasses, nitrogen-fixing legumes, and pioneer woody species indicates that spontaneous revegetation processes may substantially contribute to long-term phytostabilization and ecological recovery of technogenically disturbed landscapes.

### 4.8 Plant accumulation patterns and phytostabilization potential under metal stress

The accumulation of heavy metals in plant tissues indicates that *Helianthus annuus* L. and *Triticum aestivum* L. differed markedly in their tolerance and metal-handling strategies under contaminated soil conditions. The substantially higher biomass of *H. annuus* compared with *T. aestivum* suggests greater physiological plasticity and stress tolerance, which is consistent with previous studies describing sunflower as a promising species for phytoremediation of metal-contaminated soils because of its high biomass production and capacity to accumulate Cd, Pb, Zn, and Cu in vegetative organs (Daurov et al., 2024; Khalid et al., 2018). However, the greater metal accumulation observed in *H. annuus* cannot be explained solely by higher biomass production. The consistently higher root metal concentrations and BCF values indicate species-specific physiological mechanisms related to metal uptake, sequestration, and tolerance. The extensive root system of sunflower, together with its capacity to retain metals within belowground tissues, likely contributes to enhanced accumulation and phytostabilization performance under contaminated conditions.

The dominance of Zn accumulation in both species is consistent with its relatively high mobility and essential micronutrient function in plants. In the present study, Zn concentrations in *H. annuus* roots were more than threefold higher than in *T. aestivum*, while shoot Zn levels were approximately twofold higher. Similar root-dominated Zn accumulation has been reported for sunflower grown in industrially contaminated soils, where Zn and Cd were preferentially retained in belowground tissues rather than reproductive organs (Paulo et al., 2023). Such preferential root retention may represent an adaptive strategy that limits toxic effects in photosynthetically active tissues while maintaining essential physiological functions supported by Zn as a micronutrient.

Cadmium showed a particularly important ecological pattern. Although Cd concentrations in soils were lower than those of Zn and Cu, root Cd accumulation was high, especially in *H. annuus*. This agrees with studies showing that Cd is readily taken up by plant roots and may remain highly bioavailable even at comparatively low soil concentrations. In sunflower, Cd uptake has often been reported to exceed Pb uptake, supporting the interpretation that *H. annuus* has a relatively strong capacity for Cd accumulation under contaminated conditions (dos Santos Junior et al., 2025).

The consistently higher concentrations of Zn, Cd, Cu, and Pb in roots than in shoots indicate that both species primarily restricted upward metal transport. This root-sequestration pattern is characteristic of phytostabilization rather than efficient phytoextraction. For wheat, comparable studies have shown that heavy metals often accumulate preferentially in roots, with lower transfer to aerial tissues, particularly under contaminated or wastewater-affected soil conditions (Liu et al., 2025).

Copper and Pb exhibited comparatively lower shoot accumulation, suggesting stronger physiological regulation of their translocation. Lead, in particular, is commonly characterized by limited mobility in plants because of its strong binding to root cell walls and low xylem transport efficiency. This behavior is largely controlled by the strong affinity of Pb for pectins, phosphate groups, and extracellular binding sites within root tissues. Consequently, a substantial proportion of absorbed Pb remains immobilized within the rhizosphere and root cortex, thereby restricting long-distance transport to shoots and reducing translocation through the vascular system. The low Pb accumulation observed in both species therefore agrees with previous reports on restricted Pb transfer in wheat and sunflower systems (Shumaker and Begonia, 2005).

Overall, the observed pattern demonstrates that *H. annuus* possesses greater phytostabilization potential than *T. aestivum*, combining higher biomass production with stronger root accumulation of Zn, Cd, and Cu. However, because shoot concentrations remained considerably lower than root concentrations, these species should be interpreted primarily as root accumulators rather than efficient phytoextractors. This distinction is important for remediation planning, as root retention may reduce contaminant mobility and erosion-driven redistribution but does not necessarily imply effective removal of metals from contaminated soils.

### 4.9 Bioconcentration and translocation behavior of heavy metals in dominant agricultural crops

The BCF and TF patterns indicate that heavy metal uptake by *Helianthus annuus* and *Triticum aestivum* was governed primarily by root retention rather than efficient transfer to aerial tissues. This interpretation is supported by the consistently higher root BCF compared with shoot BCF and by TF values mostly below 1. In phytoremediation studies, TF< 1 is generally interpreted as limited metal movement from roots to shoots, while higher BCF in roots indicates a stronger phytostabilization response rather than phytoextraction (Vu Ngoc Ba et al., 2024).

The markedly higher BCF values in *H. annuus*, especially for Cu, Zn, and Cd, suggest that sunflower has greater capacity to immobilize metals in belowground tissues than wheat. This agrees with previous studies reporting that *H. annuus* can tolerate and accumulate metals in contaminated soils and is frequently considered a promising species for phytoremediation because of its high biomass and metal uptake potential (Kalyvas et al., 2022). In the present study, Cu showed the highest root BCF in sunflower, whereas Zn also demonstrated strong root accumulation, indicating that these metals were actively retained in the root system. The exceptionally high Cu BCF values observed in *H. annuus* should be interpreted in relation to both plant physiology and local geochemical conditions. Bioconcentration factors are strongly influenced by metal bioavailability and soil concentrations; therefore, elevated BCF values may occur where root uptake remains high despite comparatively moderate soil Cu concentrations. Similar patterns of enhanced Cu accumulation by sunflower have been reported in previous phytoremediation studies, highlighting the species’ capacity for effective root-level metal sequestration under contaminated conditions (De Maria and Rivelli, 2013).

Cadmium showed particularly important ecological behavior. Although Cd concentrations in soil were lower than Zn and Cu, its root BCF was high, especially in *H. annuus*. This confirms the high bioavailability and mobility of Cd in contaminated soils. Similar studies have shown that Cd can be readily absorbed by roots and may represent a major ecological concern even when its total soil concentration is comparatively lower than other metals (Usman et al., 2019).

In contrast, *T. aestivum* showed lower BCF values for most metals, indicating weaker accumulation capacity under the same contaminated conditions. This pattern is consistent with studies on wheat showing that heavy metals are often retained mainly in roots, with relatively limited transfer to shoots (Cheţan et al., 2025). The low TF values for Zn and Cd in both species further indicate strong root sequestration, which may reduce metal transfer to aboveground biomass.

Pb showed the highest TF variability, particularly in *T. aestivum*, where some values approached or exceeded 1. However, because Pb concentrations remained very low in both root and shoot tissues, this variability should be interpreted cautiously. Under low-concentration conditions, relatively small differences between root and shoot metal concentrations may produce disproportionately large changes in TF values. Therefore, the observed variability likely reflects, at least in part, mathematical sensitivity of the TF calculation rather than biologically meaningful enhancement of Pb translocation. Overall, the low absolute Pb concentrations and the absence of consistent shoot enrichment indicate that root retention remained the dominant mechanism controlling Pb behavior in both investigated species. Overall, the combined BCF and TF results suggest that *H. annuus* has stronger phytostabilization potential, while *T. aestivum* demonstrates lower metal accumulation efficiency. Therefore, the dominant plant response under mining-related contamination was root-level immobilization rather than effective phytoextraction. The importance of metal immobilization as a remediation pathway has also been demonstrated in studies employing soil amendments. For example, Yuan et al. (2026) reported that phosphorus-modified biochar effectively reduced heavy metal bioavailability and improved soil fertility through stabilization processes. Although the remediation approach differs from plant-mediated phytostabilization, both mechanisms contribute to limiting contaminant mobility and reducing ecological risk. These findings support the interpretation that root sequestration observed in the present study may represent an important pathway for long-term stabilization of heavy metals in mining-affected soils. Although the present study primarily focused on phytostabilization potential, the accumulation of heavy metals in *Helianthus annuus* and *Triticum aestivum* also has important food safety implications. Both species are widely cultivated agricultural crops, and the presence of Zn, Cu, Cd, and Pb in plant tissues may contribute to contaminant transfer through the food chain and increase potential human exposure in mining-affected agroecosystems. In particular, cadmium deserves special attention because of its high toxicity, mobility, and tendency to accumulate in biological systems. Therefore, cultivation of food crops in historically contaminated mining areas should be accompanied by regular soil and plant monitoring to minimize potential agricultural and public health risks. Although three biological replicates were used for each plant species, which is consistent with many environmental monitoring and plant accumulation studies, a larger sample size would improve statistical robustness and provide a more comprehensive assessment of spatial variability in heavy metal uptake. Therefore, the observed accumulation patterns should be interpreted as representative of the investigated conditions rather than universal responses across the entire mining area. Future studies incorporating a greater number of sampling locations, biological replicates, and seasonal observations would improve assessment of temporal variability and strengthen evaluation of long-term phytostabilization efficiency in mining-affected ecosystems.

### 4.10 Study limitations and future perspectives

Despite the robustness of the obtained results, several limitations should be acknowledged. First, the study was based on 17 sampling sites, which may not fully capture the entire spatial heterogeneity of the Belousovka mining landscape. Nevertheless, the sampling network was designed to encompass the principal contamination gradients and major landscape units within the study area. Second, the investigation was conducted during a single sampling campaign and therefore did not evaluate seasonal or interannual variations in heavy metal distribution, mobility, and plant uptake. Third, the assessment was based on total metal concentrations, whereas metal bioavailability and geochemical speciation were not directly determined through sequential extraction or related fractionation approaches. Consequently, the actual mobility and biological accessibility of metals may differ from estimates based solely on total concentrations.

Despite these limitations, the integration of soil physicochemical characterization, contamination and ecological risk indices, multivariate statistical analyses, vegetation assessment, and plant accumulation parameters provides a robust baseline evaluation of contamination status and remediation potential in the Belousovka mining district. It should be emphasized that phytostabilization does not remove heavy metals from contaminated soils but rather reduces their mobility, bioavailability, and environmental dispersion. Therefore, the long-term effectiveness of this remediation strategy depends on continuous environmental monitoring to assess the stability of immobilized contaminants and potential changes in metal mobility under changing environmental conditions.

Future investigations should incorporate larger sampling networks, long-term monitoring programs, and metal speciation analyses to improve understanding of contaminant behavior, ecological risk dynamics, and the sustainability of remediation processes in mining-affected ecosystems. In addition, the physiological, biochemical, and molecular mechanisms underlying heavy metal tolerance and retention in *Helianthus annuus* were beyond the scope of the present study. Future research should investigate gene expression patterns, antioxidant defense responses, root-associated microbial communities, and plant–soil interactions that contribute to metal immobilization and long-term remediation efficiency. Such approaches would provide a more mechanistic understanding of plant adaptation to contaminated environments and strengthen the development of sustainable nature-based remediation strategies. Furthermore, the phytostabilization assessment was limited to the dominant cultivated species present at the study sites. Comparative evaluation of additional native species, metallophytes, and naturally colonizing vegetation may provide further insights into species-specific adaptation strategies and remediation potential under mining-induced environmental stress.

## 5 Conclusion

This study demonstrated that the soils of the Belousovka mining area represent a highly heterogeneous post-mining geochemical system strongly affected by long-term polymetallic mining activity and lithological variability. Integrated assessment of soil physicochemical properties, granulometric composition, heavy metal accumulation, pollution indices, ecological risk, and plant uptake patterns revealed pronounced spatial differentiation in contamination intensity and environmental functioning across the investigated territory.

The investigated soils were predominantly characterized by silt-rich textures with variable sand contents and comparatively low clay fractions, reflecting heterogeneous sediment redistribution and mixed parent material composition. Physicochemical analyses revealed substantial variability in carbonate content, nutrient status, and exchangeable cation composition, indicating strong lithological control over soil geochemistry. Weakly acidic to near-neutral pH conditions together with elevated exchangeable Ca²^+^ concentrations suggest that carbonate-bearing substrates partially buffer acidification processes and influence heavy metal retention within the study area.

Among the investigated metals, Zn and Cu represented the dominant contaminants and showed strong localized enrichment associated with former mining and ore-processing zones. Zinc concentrations exceeded maximum permissible concentrations by more than 70-fold, while Cu exceeded regulatory thresholds by up to 68-fold. Despite lower absolute concentrations, Cd constituted the principal ecological risk factor because of its elevated toxicity and extremely high ecological risk coefficients. Pollution indices demonstrated severe spatial heterogeneity, with contamination hotspots concentrated near historical mining infrastructure, whereas peripheral soils generally exhibited lower ecological disturbance.

Multivariate analyses confirmed that heavy metal distribution and ecological risk are controlled by the interaction between technogenic contamination and soil physicochemical properties. Soil texture, carbonate content, pH, exchangeable cations, and terrain-related redistribution processes significantly influenced contaminant mobility and ecological gradients. The results additionally highlight the importance of land cover heterogeneity and terrain-controlled redistribution processes for vegetation stability and ecological functioning in post-mining landscapes.

The investigated plant species demonstrated contrasting accumulation strategies under contaminated conditions. In particular, *Helianthus annuus* L. exhibited substantially greater biomass production, root development, and metal accumulation capacity than *Triticum aestivum* L., especially for Zn, Cu, and Cd. The higher bioconcentration factors observed in *H. annuus* indicate its enhanced tolerance to contaminated substrates and its ability to maintain active physiological functioning under elevated heavy metal stress. At the same time, translocation factor values predominantly below 1 confirmed that metal transfer from roots to aboveground tissues remained restricted, suggesting that root sequestration and immobilization constituted the dominant adaptive mechanism. Such a physiological response is particularly important in contaminated mining environments because it reduces the migration of toxic elements into aerial plant organs and minimizes their entry into trophic chains.

The strong root accumulation capacity, combined with relatively high biomass productivity, indicates that *Helianthus annuus* L. possesses significant phytostabilization potential in technogenically disturbed soils. However, phytostabilization should not be regarded as a mechanism for contaminant removal, since heavy metals remain within the soil–plant system. Instead, its primary function is to reduce metal mobility, limit secondary dispersion, and decrease ecological exposure pathways. Consequently, the long-term effectiveness of phytostabilization strategies depends on continuous environmental monitoring to ensure the stability of immobilized contaminants and to evaluate potential changes in metal mobility under future environmental conditions. Its extensive root system likely contributes to substrate stabilization, reduction of wind and water erosion, and limitation of contaminated particle redistribution across the post-mining landscape. In addition, dense vegetative cover formed by *H. annuus* may improve microenvironmental conditions, promote organic matter accumulation, and support the gradual recovery of biological activity in degraded soils. These characteristics make sunflower not only a tolerant species under polymetallic stress conditions but also a promising candidate for ecological restoration strategies in post-mining environments affected by long-term ore extraction and tailing weathering. The observed adaptive responses of *Helianthus annuus* L. under polymetallic stress conditions further indicate the presence of efficient physiological tolerance mechanisms allowing the species to maintain growth and metabolic activity in contaminated substrates. Such adaptive plasticity highlights the ecological importance of tolerant pioneer vegetation in the natural recovery and long-term stabilization of technogenically transformed ecosystems. From a practical remediation perspective, the identified contamination hotspots, particularly those characterized by elevated Cd-related ecological risk, should be considered priority targets for environmental management and long-term monitoring. The demonstrated phytostabilization potential of Helianthus annuus L. suggests that this species may be incorporated into revegetation and soil stabilization programs aimed at reducing contaminant mobility, minimizing secondary dispersion of metal-enriched particles, and enhancing ecosystem recovery. Future remediation strategies for the Belousovka mining area should integrate targeted risk-based management of highly contaminated sites, phytostabilization measures, and continuous monitoring of soil and vegetation quality to support sustainable restoration of post-mining landscapes.

The dominance of perennial grasses, ruderal herbs, nitrogen-fixing legumes, and pioneer woody species within industrial areas additionally indicates active spontaneous succession and partial phytostabilization of contaminated substrates. These vegetation communities may contribute to long-term ecological stabilization and natural recovery of mining-affected ecosystems.

In contrast, *Triticum aestivum* L. demonstrated comparatively lower accumulation capacity and weaker tolerance to contaminated conditions, reflecting species-specific physiological limitations under elevated heavy metal exposure. Reduced accumulation and biomass performance suggest that wheat is more sensitive to polymetallic stress and therefore less suitable for remediation-oriented stabilization programs within heavily contaminated mining substrates. Nevertheless, the contrasting responses observed between the two species emphasize the importance of species selection in phytoremediation and ecological rehabilitation planning for technogenically transformed landscapes.

Overall, the study provides important insights into contaminant dynamics, ecological risk formation, vegetation adaptation, and plant–soil interactions in technogenically transformed ecosystems. The obtained results emphasize that vegetation plays a critical functional role not only as an indicator of ecological disturbance but also as an active regulator of contaminant stabilization and landscape recovery processes. In particular, the demonstrated tolerance and phytostabilization potential of *Helianthus annuus* L. establish a valuable scientific basis for the development of sustainable remediation approaches and future ecological restoration programs in polymetallic post-mining regions.

## Data Availability

The original contributions presented in the study are included in the article/**Supplementary Material**. Further inquiries can be directed to the corresponding author.
