# Supplementary material for: Integrated assessment of heavy metal dynamics, ecological risk, and plant-mediated phytostabilization in mining-affected soils
Source: Front Plant Sci. 2026 Jul 9;17:1894392. doi: 10.3389/fpls.2026.1894392 (PMC13391431; doi:10.3389/fpls.2026.1894392)
Supplement: Supplementary file 1 [file SupplementaryFile1.docx]

Supplementary Material

# Supplementary Data

Supplementary Material should be uploaded separately on submission. Please include any supplementary data, figures and/or tables.

Supplementary material is not typeset so please ensure that all information is clearly presented, the appropriate caption is included in the file and not in the manuscript, and that the style conforms to the rest of the article.

# Supplementary Figures and Tables

For more information on Supplementary Material and for details on the different file types accepted, please see [here](https://www.frontiersin.org/guidelines/author-guidelines#supplementary-material).

## Supplementary Figures


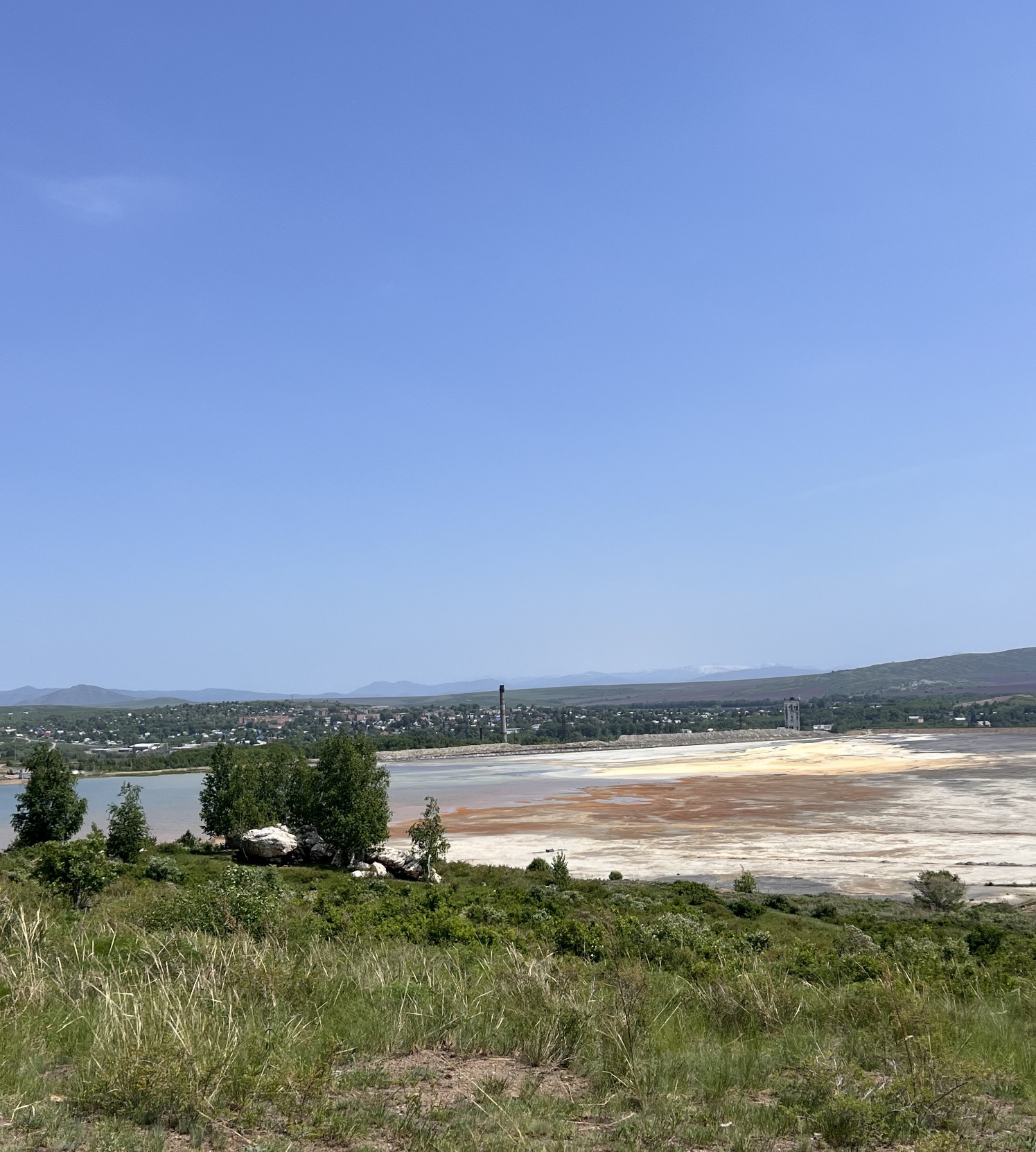


**Supplementary Figure 1.** Study area in the Belousovka industrial region (East Kazakhstan Region)

**Supplementary Tables**

**Supplementary Table 1.** Geographic coordinates and environmental characteristics of the soil sampling sites

| **Sample** | **Latitude** | **Longitude** | **Mesotopography** | **Soil type** **(WRB)** | **Functional zone** |
| --- | --- | --- | --- | --- | --- |
| S1 | 50.12997 | 82.51746 | gentle slope | Haplic Chernozem | industrial zone |
| S2 | 50.13180 | 82.51534 | gentle slope | Haplic Chernozem | industrial zone |
| S3 | 50.13008 | 82.51066 | river terrace | Haplic Fluvisol | industrial zone |
| S4 | 50.12734 | 82.50851 | interfluve | Haplic Chernozem | industrial zone |
| S5 | 50.12455 | 82.50509 | gentle slope | Haplic Chernozem | industrial zone |
| S6 | 50.12392 | 82.51152 | river terrace | Haplic Fluvisol | industrial zone |
| S7 | 50.11832 | 82.50514 | slope | Haplic Chernozem | industrial zone |
| S8 | 50.12204 | 82.49545 | river terrace | Haplic Fluvisol | industrial zone |
| S9 | 50.11539 | 82.48662 | interfluve | Haplic Chernozem | industrial zone |
| S10 | 50.13000 | 82.47115 | slope | Haplic Chernozem | industrial zone |
| S11 | 50.13005 | 82.48750 | gentle slope | Haplic Chernozem | industrial zone |
| S12 | 50.13718 | 82.49327 | river terrace | Haplic Fluvisol | industrial zone |
| S13 | 50.13483 | 82.50080 | interfluve | Haplic Chernozem | agricultural zone |
| S14 | 50.13813 | 82.50620 | gentle slope | Haplic Chernozem | agricultural zone |
| S15 | 50.13438 | 82.50462 | river terrace | Haplic Fluvisol | agricultural zone |
| S16 | 50.12997 | 82.51746 | gentle slope | Haplic Chernozem | control zone |
| S17 | 50.13180 | 82.51534 | gentle slope | Haplic Chernozem | control zone |

Supplementary Table 2. LOD and LOQ values and linear parameters for the HM calibration standards

| **HM** | **R2** | **Calibration Curve**  **Equation** | **Range (mg·L⁻¹)** | **LOD**  **(mg·L⁻¹)** | **LOQ**  **(mg·L⁻¹)** |
| --- | --- | --- | --- | --- | --- |
| Pb | 0.9996 | y = 0.9824x − 0.1931 | 1.0-10.0 | 0.05 | 0.15 |
| Zn | 0.9997 | y = 1.0128x − 0.0876 | 1.0-10.0 | 0.08 | 0.24 |
| Cd | 0.9995 | y = 1.1789x − 0.1024 | 1.0-10.0 | 0.04 | 0.12 |
| Cu | 0.9996 | y = 0.9457x − 0.0642 | 1.0-10.0 | 0.07 | 0.21 |

Note: R, correlation coefficient; LOD, limit of detection; LOQ, limit of quantification

Supplementary Table 3. Reference standard certified and measured values and the recovery.

| **HM** | **Certified values (mg·L⁻¹)** | **Measured values (mg·L⁻¹)** | **Recovery**  **(%)** | **Mean recovery ± SD (%)** |
| --- | --- | --- | --- | --- |
| Pb | 5.0 | 4.98 | 99.2 | 99.4 ± 0.32 |
|  | 10.0 | 9.95 | 99.5 |  |
| Zn | 5.0 | 4.92 | 98.4 | 99.1 ± 1.08 |
|  | 10.0 | 9.97 | 99.7 |  |
| Cd | 5.0 | 5.03 | 100.6 | 100.3 ± 0.41 |
|  | 10.0 | 10.01 | 100.1 |  |
| Cu | 5.0 | 4.98 | 99.8 | 100.1 ± 0.36 |
|  | 10.0 | 10.04 | 100.4 |  |

Note: Data are means ± standard deviations

**Supplementary Table S4.** Classification of Soil Contamination Levels Based on the Geoaccumulation Index (Igeo).

| **Class I_geo_** | **Grade I_geo_** | **Pollution Grade** |
| --- | --- | --- |
| Class 0 | Igeo ≤ 0 | uncontaminated |
| Class 1 | 0 < Igeo ≤ 1 | uncontaminated to moderately contaminated |
| Class 2 | 1 < Igeo ≤ 2 | moderately contaminated |
| Class 3 | 2 < Igeo ≤ 3 | moderately to heavily contaminated |
| Class 4 | 3 < Igeo ≤ 4 | heavily contaminated |
| Class 5 | 4 < Igeo ≤ 5 | heavily to extremely contaminated |
| Class 6 | Igeo > 5 | extremely contaminated |

Supplementary Table S5. Interpretation of the value of the potential environmental risk indicator.

| **Er** | **Levels** | **Ri** | **Levels** |
| --- | --- | --- | --- |
| Er ˂ 40 | Low | RI ˂ 150 | Low |
| 40 ≤ Er ˂ 80 | Moderate | 150 ≤ RI ˂ 300 | Moderate |
| 80 ≤ Er ˂ 160 | Considerable | 300 ≤ RI ˂ 600 | Considerable |
| 160 ≤ Er ˂ 320 | High | RI ≥ 600 | Very high |
| Er ≥ 320 | Very high |  |  |

Note: RI is the comprehensive potential ecological risk index; Er is the individual potential ecological risk index of heavy metal.

**Supplementary Table** **S6.** Physicochemical properties of soils at the sampling sites

| **Sampling site** | **Total organic carbon, %** | **Available nitrogen (N), mg·kg⁻¹** | **Available P₂O₅ (mg·kg⁻¹)** | **Exchangeable K₂O (mg·kg⁻¹)** | **рН** | **СaCO_3_, (%)** | **Exchangeable base cations (mmol(+)/kg)** | | | | |
| --- | --- | --- | --- | --- | --- | --- | --- | --- | --- | --- | --- |
|  |  |  |  |  |  |  | **Са^2+^** | | **Mg^2+^** | **Na^+^** | **K^+^** |
| S1 | 2.89 | 61.6 | 16 | 250 | 7.08 | 1.68 | | 222.7 | 29.7 | 2.3 | 1.8 |
| S2 | 4.51 | 36.4 | 18 | 120 | 6.81 | 1.2 | | 173.2 | 128.7 | 0.7 | 1.1 |
| S3 | 4.19 | 92.4 | 110 | 300 | 7.32 | 0.95 | | 227.7 | 138.6 | 26.2 | 1.2 |
| S4 | 2.37 | 56.0 | 18 | 130 | 6.86 | 29.49 | | 148.5 | 34.6 | 2.3 | 1.4 |
| S5 | 3.83 | 61.6 | 30 | 280 | 6.54 | 0.79 | | 54.4 | 14.8 | 2.3 | 1.0 |
| S6 | 3.06 | 42.0 | 216 | 640 | 7.18 | 4.86 | | 148.5 | 34.6 | 2.3 | 8.6 |
| S7 | 1.93 | 61.6 | 14 | 250 | 6.13 | 1.27 | | 202.9 | 84.1 | 2.3 | 1.4 |
| S8 | 2.75 | 61.6 | 22 | 100 | 6.96 | 1.27 | | 208.5 | 92.1 | 0.3 | 1.1 |
| S9 | 1.86 | 64.4 | 8 | 330 | 6.83 | 0.79 | | 203.7 | 48.5 | 2.3 | 1.4 |
| S10 | 2.09 | 47.6 | 14 | 240 | 6.55 | BDL | | 203.7 | 53.3 | 2.3 | 1.4 |
| S11 | 2.52 | 39.2 | 10 | 270 | 5.79 | BDL | | 189.1 | 38.8 | 2.3 | 1.4 |
| S12 | 2.52 | 50.4 | 14 | 210 | 6.84 | 2.45 | | 237.6 | 24.2 | 2.3 | 1.4 |
| S13 | 1.83 | 33.6 | 12 | 140 | 5.74 | 0.77 | | 184.3 | 38.8 | 2.3 | 1.4 |
| S14 | 2.02 | 39.2 | 10 | 210 | 6.18 | 1.02 | | 208.5 | 43.6 | 2.3 | 1.4 |
| S15 | 4.22 | 53.2 | 12 | 210 | 6.41 | 1.43 | | 232.8 | 58.2 | 2.3 | 1.0 |
| S16 | 2.23 | 47.6 | 16 | 240 | 6.3 | 0.77 | | 232.8 | 53.3 | 2.3 | 1.2 |
| S17 | 5.17 | 75.6 | 48 | 160 | 4.99 | 0.39 | | 295.8 | 72.7 | 2.0 | 0.9 |

Note: BDL (Below Detection Limit)

**Supplementary Table S7.** Particle-size distribution, USDA texture classes, and Kachinsky granulometric classification of the studied soil samples

| **Sampling Site** | **Fraction Content (% of Absolute Dry Soil)** | | | **USDA**  **texture class** | **Fractions <0.01 mm (%)** | **Kachinsky classification** |
| --- | --- | --- | --- | --- | --- | --- |
|  | **Particle Size (mm)** | | |  |  |  |
|  | **Sand** | **Silt** | **Clay** |  |  |  |
| S1 | 18.99 | 69.15 | 11.87 | Silt loam | 50.33 | Light clay |
| S2 | 60.02 | 39.17 | 0.82 | Sandy loam | 6.12 | Loose sand |
| S3 | 10.76 | 83.49 | 5.76 | Silt | 27.56 | Light loam |
| S4 | 51.36 | 47.41 | 1.23 | Sandy loam | 10.22 | Coherent sand |
| S5 | 40.71 | 55.61 | 3.68 | Silt loam | 25.35 | Light loam |
| S6 | 11.18 | 78.63 | 10.19 | Silt loam | 31.37 | Medium loam |
| S7 | 0.93 | 89.62 | 9.46 | Silt | 39.47 | Medium loam |
| S8 | 13.62 | 83.92 | 2.47 | Silt | 30.85 | Medium loam |
| S9 | 7.91 | 84.69 | 7.40 | Silt | 32.48 | Medium loam |
| S10 | 15.83 | 76.37 | 7.80 | Silt loam | 37.36 | Medium loam |
| S11 | 53.53 | 40.77 | 5.71 | Sandy loam | 13.05 | Coherent sand |
| S12 | 16.48 | 77.35 | 6.17 | Silt loam | 42.38 | Heavy loam |
| S13 | 49.06 | 46.45 | 4.48 | Sandy loam | 30.97 | Medium loam |
| S14 | 58.84 | 30.87 | 10.29 | Sandy loam | 38.69 | Medium loam |
| S15 | 13.42 | 85.35 | 1.23 | Silt | 22.98 | Light loam |
| S16 | 16.96 | 68.24 | 14.80 | Silt loam | 49.74 | Heavy loam |
| S17 | 23.65 | 70.16 | 6.19 | Silt loam | 32.60 | Medium loam |

**Supplementary Table** **S8.** Concentrations of heavy metals in soils affected by mining activities in Belousovka, East Kazakhstan

| **Sampling site** | **Zn, mg·kg⁻¹** | **Сu, mg·kg⁻¹** | **Сd, mg·kg⁻¹** | **Pb, mg·kg⁻¹** |
| --- | --- | --- | --- | --- |
| S1 | 4.2 ± 1.1 | 0.8 ± 0.5 | 0.5 ± 0.4 | 1.1 ± 0.8 |
| S2 | 565 ± 2.8 | 57 ± 1.7 | 5.5 ± 1.2 | 8.7 ± 2.1 |
| S3 | 195 ± 2.3 | 4.4 ± 1.0 | 3.0 ± 0.9 | 2.8 ± 1.1 |
| S4 | 1641 ± 3.2 | 205 ± 2.6 | 31 ± 1.8 | 10 ± 2.3 |
| S5 | 398 ± 2.1 | 17.8 ± 1.4 | 4.2 ± 1.1 | 3.2 ± 0.9 |
| S6 | 112 ± 1.9 | 5.7 ± 1.0 | 2.7 ± 0.8 | 1.8 ± 0.7 |
| S7 | 12.1 ± 1.0 | 0.3 ± 0.2 | 0.7 ± 0.4 | 0.6 ± 0.3 |
| S8 | 47 ± 2.0 | 9.2 ± 1.3 | 1.0 ± 0.5 | 1.2 ± 0.6 |
| S9 | 0.9 ± 0.3 | 1.6 ± 0.7 | 0.5 ± 0.2 | 0.3 ± 0.1 |
| S10 | 5.7 ± 0.9 | 1.3 ± 0.6 | 1.0 ± 0.5 | 1.0 ± 0.4 |
| S11 | 5.8 ± 1.1 | 1.4 ± 0.5 | 0.5 ± 0.3 | 0.7 ± 0.2 |
| S12 | 11.5 ± 1.4 | 1.3 ± 0.6 | 0.6 ± 0.4 | 0.4 ± 0.2 |
| S13 | 2.1 ± 0.8 | 0.8 ± 0.4 | 0.4 ± 0.2 | 0.7 ± 0.3 |
| S14 | 4.2 ± 1.0 | 0.6 ± 0.3 | 1.0 ± 0.5 | 1.0 ± 0.4 |
| S15 | 5.9 ± 1.2 | 1.1 ± 0.5 | 1.1 ± 0.6 | 0.7 ± 0.3 |
| S16 | 1.7 ± 0.5 | 1.8 ± 0.7 | 0.5 ± 0.2 | 1.2 ± 0.5 |
| S17 | 4.0 ± 0.9 | 1.1 ± 0.4 | 0.2 ± 0.1 | 0.7 ± 0.2 |

Note: Data are means ± standard deviations.

**Supplementary Table** **S9.** Heavy metal concentrations and ecological risk assessment of soils affected by mining activities in Belousovka, East Kazakhstan Region

| **Sampling site** | **Zn** | **Cu** | **Cd** | **Pb** | **PLI** | **RI** |
| --- | --- | --- | --- | --- | --- | --- |
| S1 | 4.2 ± 1.1 | 0.8 ± 0.5 | 0.5 ± 0.4 | 1.1 ± 0.8 | 1.08 | 52.88 |
| S2 | 565 ± 2.8 | 57 ± 1.7 | 5.5 ± 1.2 | 8.7 ± 2.1 | 57.91 | 912.02 |
| S3 | 195 ± 2.3 | 4.4 ± 1.0 | 3.0 ± 0.9 | 2.8 ± 1.1 | 8.49 | 355.47 |
| S4 | 1641 ± 3.2 | 205 ± 2.6 | 31 ± 1.8 | 10 ± 2.3 | 74.67 | 3992.46 |
| S5 | 398 ± 2.1 | 17.8 ± 1.4 | 4.2 ± 1.1 | 3.2 ± 0.9 | 16.24 | 577.87 |
| S6 | 112 ± 1.9 | 5.7 ± 1.0 | 2.7 ± 0.8 | 1.8 ± 0.7 | 6.9 | 299.86 |
| S7 | 12.1 ± 1.0 | 0.3 ± 0.2 | 0.7 ± 0.4 | 0.6 ± 0.3 | 1.03 | 68.44 |
| S8 | 47 ± 2.0 | 9.2 ± 1.3 | 1.0 ± 0.5 | 1.2 ± 0.6 | 4.4 | 140.25 |
| S9 | 0.9 ± 0.3 | 1.6 ± 0.7 | 0.5 ± 0.2 | 0.3 ± 0.1 | 0.63 | 50.27 |
| S10 | 5.7 ± 0.9 | 1.3 ± 0.6 | 1.0 ± 0.5 | 1.0 ± 0.4 | 1.52 | 97.46 |
| S11 | 5.8 ± 1.1 | 1.4 ± 0.5 | 0.5 ± 0.3 | 0.7 ± 0.2 | 1.2 | 53.4 |
| S12 | 11.5 ± 1.4 | 1.3 ± 0.6 | 0.6 ± 0.4 | 0.4 ± 0.2 | 1.27 | 62.05 |
| S13 | 2.1 ± 0.8 | 0.8 ± 0.4 | 0.4 ± 0.2 | 0.7 ± 0.3 | 0.76 | 41.47 |
| S14 | 4.2 ± 1.0 | 0.6 ± 0.3 | 1.0 ± 0.5 | 1.0 ± 0.4 | 1.16 | 94.52 |
| S15 | 5.9 ± 1.2 | 1.1 ± 0.5 | 1.1 ± 0.6 | 0.7 ± 0.3 | 1.38 | 103.83 |
| S16 | 1.7 ± 0.5 | 1.8 ± 0.7 | 0.5 ± 0.2 | 1.2 ± 0.5 | 1.08 | 55.98 |
| S17 | 4.0 ± 0.9 | 1.1 ± 0.4 | 0.2 ± 0.1 | 0.7 ± 0.2 | 0.94 | 26.02 |

Note: Data are means ± standard deviations.

**Supplementary Table** **S10.** Contamination factor (Cf), geoaccumulation index (Igeo), and ecological risk factor (Er) of heavy metals in soils of the Belousovka area, East Kazakhstan Region

| **Sampling site** | **Zn** | | | **Cu** | | | **Cd** | | | **Pb** | | |
| --- | --- | --- | --- | --- | --- | --- | --- | --- | --- | --- | --- | --- |
|  | **C_f_** | **I_geo_** | **Eᵣ** | **C_f_** | **I_geo_** | **Eᵣ** | **C_f_** | **I_geo_** | **Eᵣ** | **C_f_** | **I_geo_** | **Eᵣ** |
| S1 | 1.47 | -0.03 | 1.47 | 0.55 | -1.44 | 2.76 | 1.43 | -0.07 | 42.86 | 1.16 | -0.38 | 5.79 |
| S2 | 198.25 | 7.05 | 198.25 | 39.31 | 4.71 | 196.55 | 15.71 | 3.39 | 471.43 | 9.16 | 2.61 | 45.79 |
| S3 | 68.42 | 5.51 | 68.42 | 3.03 | 1.02 | 15.17 | 8.57 | 2.51 | 257.14 | 2.95 | 0.97 | 14.74 |
| S4 | 575.79 | 8.58 | 575.79 | 141.38 | 6.56 | 706.9 | 88.57 | 5.88 | 2657.14 | 10.53 | 2.81 | 52.63 |
| S5 | 139.65 | 6.54 | 139.65 | 12.28 | 3.03 | 61.38 | 12 | 3 | 360 | 3.37 | 1.17 | 16.84 |
| S6 | 39.3 | 4.71 | 39.3 | 3.93 | 1.39 | 19.66 | 7.71 | 2.36 | 231.43 | 1.89 | 0.33 | 9.47 |
| S7 | 4.25 | 1.5 | 4.25 | 0.21 | -2.28 | 1.03 | 2 | 0.42 | 60 | 0.63 | -1.25 | 3.16 |
| S8 | 16.49 | 3.46 | 16.49 | 6.34 | 2.08 | 31.72 | 2.86 | 0.93 | 85.71 | 1.26 | -0.25 | 6.32 |
| S9 | 0.32 | -2.25 | 0.32 | 1.1 | -0.45 | 5.52 | 1.43 | -0.07 | 42.86 | 0.32 | -2.25 | 1.58 |
| S10 | 2 | 0.42 | 2 | 0.9 | -0.74 | 4.48 | 2.86 | 0.93 | 85.71 | 1.05 | -0.51 | 5.26 |
| S11 | 2.04 | 0.44 | 2.04 | 0.97 | -0.64 | 4.83 | 1.43 | -0.07 | 42.86 | 0.74 | -1.03 | 3.68 |
| S12 | 4.04 | 1.43 | 4.04 | 0.9 | -0.74 | 4.48 | 1.71 | 0.19 | 51.43 | 0.42 | -1.83 | 2.11 |
| S13 | 0.74 | -1.03 | 0.74 | 0.55 | -1.44 | 2.76 | 1.14 | -0.4 | 34.29 | 0.74 | -1.03 | 3.68 |
| S14 | 1.47 | -0.03 | 1.47 | 0.41 | -1.86 | 2.07 | 2.86 | 0.93 | 85.71 | 1.05 | -0.51 | 5.26 |
| S15 | 2.07 | 0.46 | 2.07 | 0.76 | -0.98 | 3.79 | 3.14 | 1.07 | 94.29 | 0.74 | -1.03 | 3.68 |
| S16 | 0.6 | -1.33 | 0.6 | 1.24 | -0.23 | 6.21 | 1.43 | -0.07 | 42.86 | 1.26 | -0.25 | 6.32 |
| S17 | 1.4 | -0.1 | 1.4 | 0.76 | -0.98 | 3.79 | 0.57 | -1.39 | 17.14 | 0.74 | -1.03 | 3.68 |

**Supplementary Table** **S11.** Ecological importance of plant species at the control site and the industrial area

| **Family** | **Species** | **Important Value** | **Projective cover (%)** | **Classification** | **Control** | **Industrial site** |
| --- | --- | --- | --- | --- | --- | --- |
| *Betulaceae* | *Betula pendula* Roth | 0.118 | 3.3 | Perennial woody pioneer | – | + |
|  | *Betula pubescens* Ehrh. | 0.073 | 2.0 | Perennial woody species | + | – |
| *Salicaceae* | *Populus nigra* L. | 0.087 | 2.4 | Perennial woody pioneer | – | + |
|  | *Populus balsamifera* L. | 0.093 | 2.6 | Perennial woody pioneer | – | + |
|  | *Salix cinerea* L. | 0.065 | 1.8 | Perennial hygrophytic shrub | – | + |
| *Rosaceae* | *Padus avium* Mill. | 0.070 | 1.9 | Perennial woody species | + | – |
|  | *Agrimonia pilosa* Ledeb. | 0.059 | 1.6 | Perennial meadow species | + | – |
|  | *Potentilla chrysantha* Trevir. | 0.057 | 1.6 | Perennial meadow species | – | + |
| *Poaceae* | *Bromus inermis* Leyss. | 0.121 | 3.4 | Perennial meadow grass | + | + |
|  | *Dactylis glomerata* L. | 0.129 | 3.6 | Perennial phytostabilizing grass | + | + |
|  | *Festuca* sp. | 0.084 | 2.3 | Perennial meadow grass | – | + |
|  | *Calamagrostis epigeios* (L.) Roth | 0.138 | 3.8 | Perennial ruderal grass | – | + |
|  | *Elytrigia repens* (L.) Nevski | 0.126 | 3.5 | Perennial rhizomatous grass | – | + |
| *Asteraceae* | *Artemisia absinthium* L. | 0.119 | 3.3 | Perennial ruderal species | + | + |
|  | *Artemisia vulgaris* L. | 0.142 | 3.9 | Perennial metal-tolerant ruderal | + | + |
|  | *Artemisia dracunculus* L. | 0.067 | 1.9 | Perennial steppe species | + | – |
|  | *Cichorium intybus* L. | 0.116 | 3.2 | Perennial ruderal species | + | + |
|  | *Carduus crispus* L. | 0.064 | 1.8 | Annual or perennial ruderal | + | – |
|  | *Echinops tricholepis* Schrenk | 0.094 | 2.6 | Perennial xerophytic species | + | + |
|  | *Cirsium vulgare* (Savi) Ten. | 0.092 | 2.5 | Annual or perennial ruderal | + | + |
|  | *Arctium tomentosum* Mill. | 0.063 | 1.7 | Biennial nitrophilous species | – | + |
|  | *Sonchus arvensis* L. | 0.066 | 1.8 | Perennial ruderal species | + | – |
|  | *Inula helenium* L. | 0.061 | 1.7 | Perennial meadow species | + | – |
|  | *Galatella* sp. | 0.056 | 1.5 | Perennial steppe species | + | – |
|  | *Tussilago farfara* L. | 0.095 | 2.6 | Perennial pioneer species | – | + |
|  | *Achillea millefolium* L. | 0.089 | 2.5 | Perennial meadow-ruderal species | – | + |
| *Fabaceae* | *Trifolium hybridum* L. | 0.108 | 3.0 | Perennial nitrogen-fixing species | + | + |
|  | *Vicia sepium* L. | 0.122 | 3.4 | Perennial meadow legume | + | + |
|  | *Melilotus albus* Medik. | 0.110 | 3.0 | Biennial nitrogen-fixing species | + | + |
|  | *Melilotus officinalis* (L.) Pall. | 0.087 | 2.4 | Biennial ruderal legume | + | – |
|  | *Medicago falcata* L. | 0.112 | 3.1 | Perennial nitrogen-fixing species | + | + |
| *Brassicaceae* | *Bunias orientalis* L. | 0.131 | 3.6 | Perennial disturbance indicator | + | – |
|  | *Armoracia rusticana* Gaertn., Mey. et Scherb. | 0.058 | 1.6 | Perennial hygrophytic species | – | + |
| *Cannabaceae* | *Humulus lupulus* L. | 0.058 | 1.6 | Perennial climbing species | – | + |
|  | *Cannabis ruderalis* Janisch | 0.069 | 1.9 | Annual ruderal species | + | – |
| *Sapindaceae* | *Acer negundo* L. | 0.082 | 2.3 | Perennial woody species | + | + |
| *Caprifoliaceae* | *Lonicera tatarica* L. | 0.061 | 1.7 | Perennial shrub | + | – |
| *Hypericaceae* | *Hypericum perforatum* L. | 0.062 | 1.7 | Perennial meadow species | – | + |
| *Amaranthaceae* | *Chenopodium album* L. | 0.074 | 2.1 | Annual ruderal species | + | – |
| *Convolvulaceae* | *Convolvulus arvensis* L. | 0.127 | 3.5 | Perennial ruderal species | + | + |
| *Euphorbiaceae* | *Euphorbia* sp. | 0.058 | 1.6 | Perennial ruderal species | – | + |
| *Scrophulariaceae* | *Verbascum songaricum* Schrenk | 0.060 | 1.7 | Biennial xerophytic species | – | + |
| *Apiaceae* | *Angelica decurrens* (Ledeb.) B. Fedtsch. | 0.057 | 1.6 | Perennial hygrophytic species | – | + |
| *Plantaginaceae* | *Plantago major* L. | 0.097 | 2.7 | Perennial disturbance indicator | – | + |
| *Malvaceae* | *Lavatera thuringiaca* L. | 0.060 | 1.7 | Perennial meadow species | + | – |
| *Boraginaceae* | *Echium vulgare* L. | 0.071 | 2.0 | Biennial ruderal species | – | + |
| *Polygonaceae* | *Rumex confertus* Willd. | 0.072 | 2.0 | Perennial nitrophilous species | – | + |
